# Supplementary material for: Secondary Metabolites and Their Cytotoxic Activity of Artemisia nitrosa Weber. and Artemisia marschalliana Spreng
Source: Molecules. 2022 Nov 21;27(22):8074. doi: 10.3390/molecules27228074 (PMC9694440; doi:10.3390/molecules27228074)
Supplement: Supplementary file 1 [file molecules-27-08074-s001.zip › molecules-1960828-supplementary.pdf]

Supplementary information.

Secondary Metabolites and Their Cytotoxic Activity of *Artemisia nitrosa* Weber. and *Artemisia marschalliana* Spreng.

Aizhan Kazymbetova [1,2,3], Magzhan Amangeldi [1,2,3], Aliya Nurlybekova [1,4], Ulpan Amzeyeva[1,4], Kunbike Baktybala [1,4], Chun-Ping Tang [3], Chang-Qiang Ke [3], Sheng Yao [3], Yang Ye [2,3 *], Janar Jenis[1, 2, 3, 4 *]

[1] The Research Center for Medicinal Plants, Al-Farabi Kazakh National University, al-Farabi ave. 71, Almaty, 050040, Kazakhstan

[2] University of Chinese Academy of Sciences, Beijing 100049, China

[3] State Key Laboratory of Drug Research, Shanghai Institute of Materia Medica,

Chinese Academy of Sciences, Shanghai 201203, China

[4] Research Institute for Natural Products & Technology, Almaty 050046, Kazakhstan

*Correspondence: yye@simm.ac.cn (Y.Y.); janarjenis@kaznu.kz (J.J.);

Content

Supplementary data for *Artemisia nitrosa* 3

Figure S1. ^{1}H NMR, ^{13}C NMR and DEPT spectrum of **1** in CDCl3. 3

Figure S2. ^{1}H NMR, ^{13}C NMR and DEPT spectrum of **2** in CD3OD 4

Figure S3. ^{1}H NMR, ^{13}C NMR and DEPT spectrum of **3** in CDCl3. 5

Figure S4. ^{1}H NMR, ^{13}C NMR and DEPT spectrum of **4** in CDCl3. 6

Figure S5. ^{1}H NMR, ^{13}C NMR and DEPT spectrum of **5** in CDCl3. 7

Figure S6. ^{1}H NMR, ^{13}C NMR and DEPT spectrum of **6** in CDCl3. 8

Figure S7. ^{1}H NMR, ^{13}C NMR and DEPT spectrum of **7** in CDCl3. 9

Figure S8. ^{1}H NMR, ^{13}C NMR and DEPT spectrum of **8** in CDCl3. 10

Figure S9. ^{1}H NMR, ^{13}C NMR and DEPT spectrum of **9** in CD3OD 11

Figure S10. ^{1}H NMR, ^{13}C NMR and DEPT spectrum of **10** in CDCl3 12

Figure S11. ^{1}H NMR, ^{13}C NMR, DEPT, HSQC, HMBC and ^{1}H-^{1}H COSY spectra of **11** in CDCl3. 13

Figure S12. UV spectrum of **11** 16

Figure S13. TOFMS spectral analysis of **11** 17

Figure S14. ^{1}H NMR, ^{13}C NMR and DEPT spectrum of **12** in CDCl3 18

Supplementary data for *Artemisia marschalliana* 19

Figure S15. ^{1}H NMR, ^{13}C NMR, DEPT, HSQC, HMBC and ^{1}H-^{1}H COSY spectra of **1'** in CDCl3. 19

Figure S16. UV spectrum of **1'** 22

Figure S17. ESI Mass spectrum of **1'** 22

Figure S18. ^{1}H NMR, ^{13}C NMR and DEPT spectrum of **2'** in Acetone d-6 23

Figure S19. ^{1}H NMR, ^{13}C NMR and DEPT spectrum of **3'** in CDCl3 24

Figure S20. ^{1}H NMR, ^{13}C NMR and DEPT spectrum of **4'** in CDCl3 25

Figure S21. ^{1}H NMR, ^{13}C NMR and DEPT spectrum of **5'** in CDCl3 26

Figure S22. ^{1}H NMR, ^{13}C NMR and DEPT spectrum of **6'** in CDCl3 27

Figure S23. ^{1}H NMR, ^{13}C NMR and DEPT spectrum of **7'** in CDCl3 28

Figure S24. ^{1}H NMR, ^{13}C NMR and DEPT spectrum of **8'** in CDCl3 29

Figure S25. ^{1}H NMR, ^{13}C NMR and DEPT spectrum of **9'** in CDCl3 30

Figure S26. ^{1}H NMR, ^{13}C NMR and DEPT spectrum of **10'** in CDCl3 31

Figure S27. ^{1}H NMR, ^{13}C NMR and DEPT spectrum of **11'** in CDCl3 32

Figure S28. ^{1}H NMR, ^{13}C NMR and DEPT spectrum of **12'** in CDCl3 33

Figure S29. ^{1}H NMR, ^{13}C NMR and DEPT spectrum of **13'** in CDCl3 34

Figure S30. Inhibitory Effects of *A.nitrosa* compounds on LPS-Enhanced Inflammatory Mediators ____ 35

Figure S31. Picture of the whole plant of *Artemisia nitrosa* 36

Figure S32. Picture of the whole plant of *Artemisia marschalliana* 36

Supplementary data for *Artemisia nitrosa*

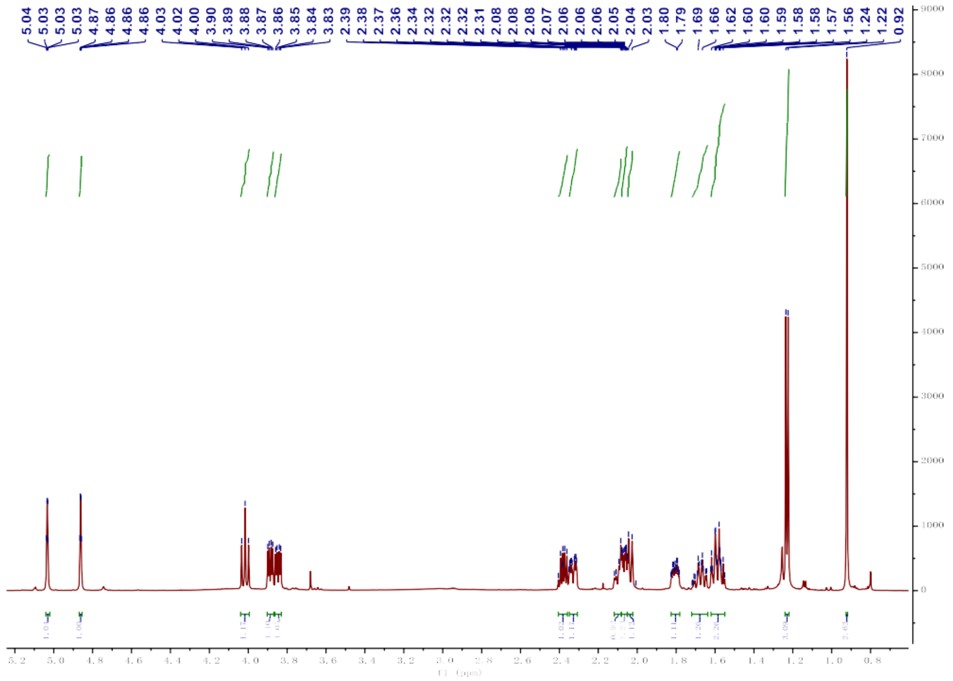

^{1}H NMR spectrum of compound **1** $CDCl_3$

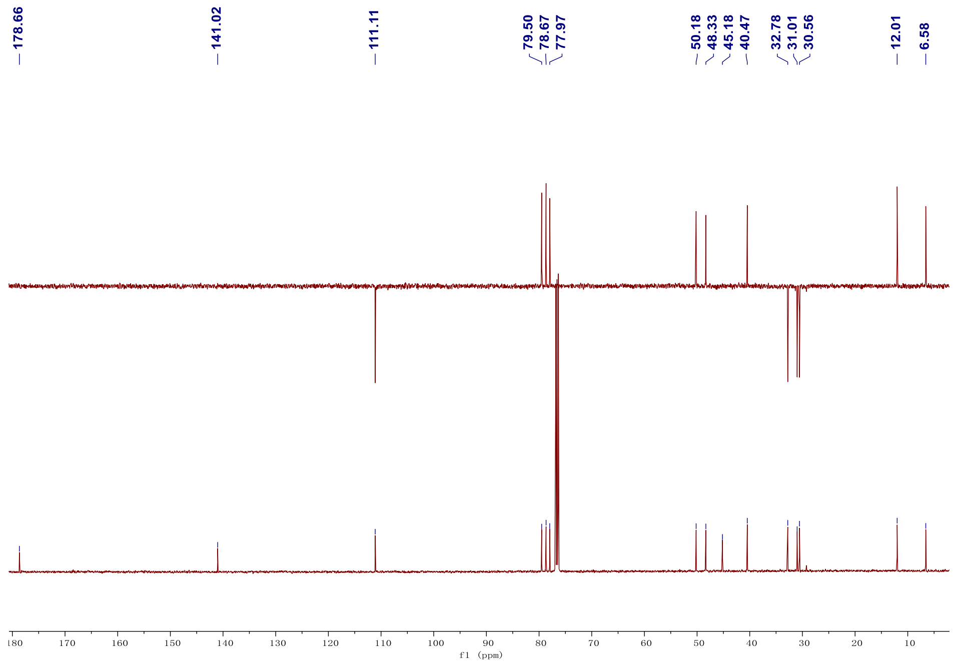

^{13}C NMR, DEPT spectrum of **1** in $CDCl_3$

Figure S1. ^{1}H NMR, ^{13}C NMR, DEPT spectrum of **1** in $CDCl_3$

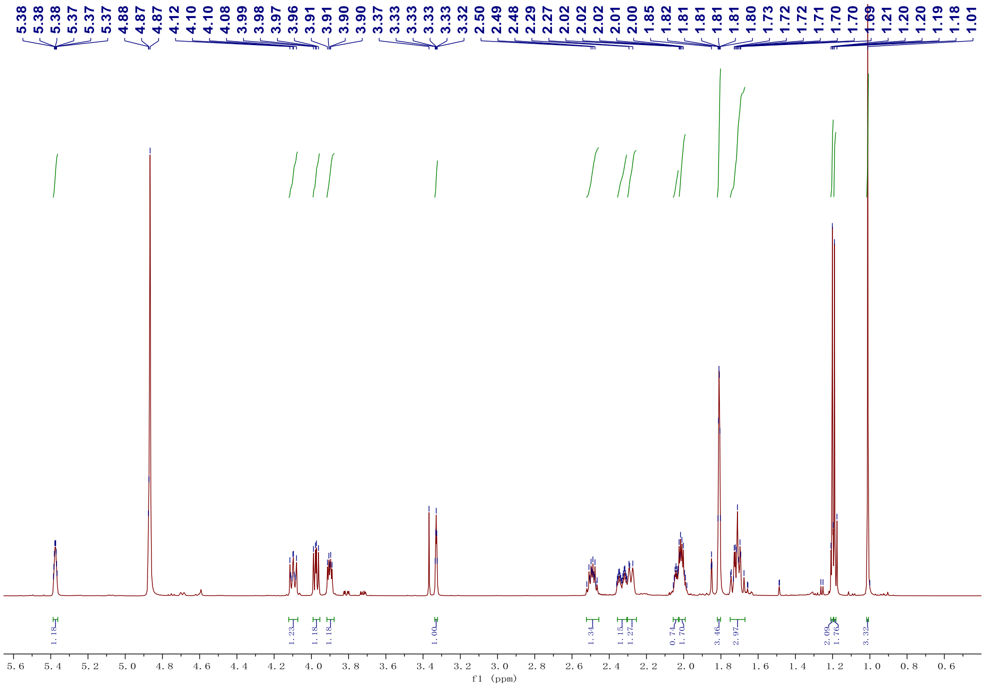

^{1}H NMR spectrum of **2** in CD_3OD.

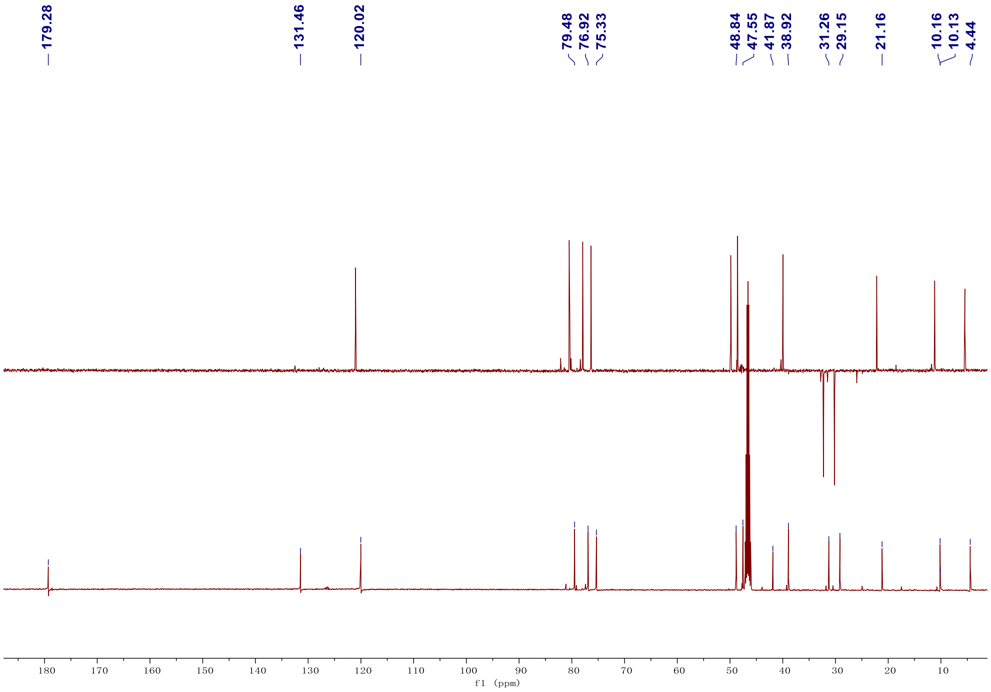

^{13}C NMR, DEPT spectrum of **2** in CD_3OD.

Figure S2. ^{1}H NMR, ^{13}C NMR, DEPT spectrum of **2** in CD_3OD.

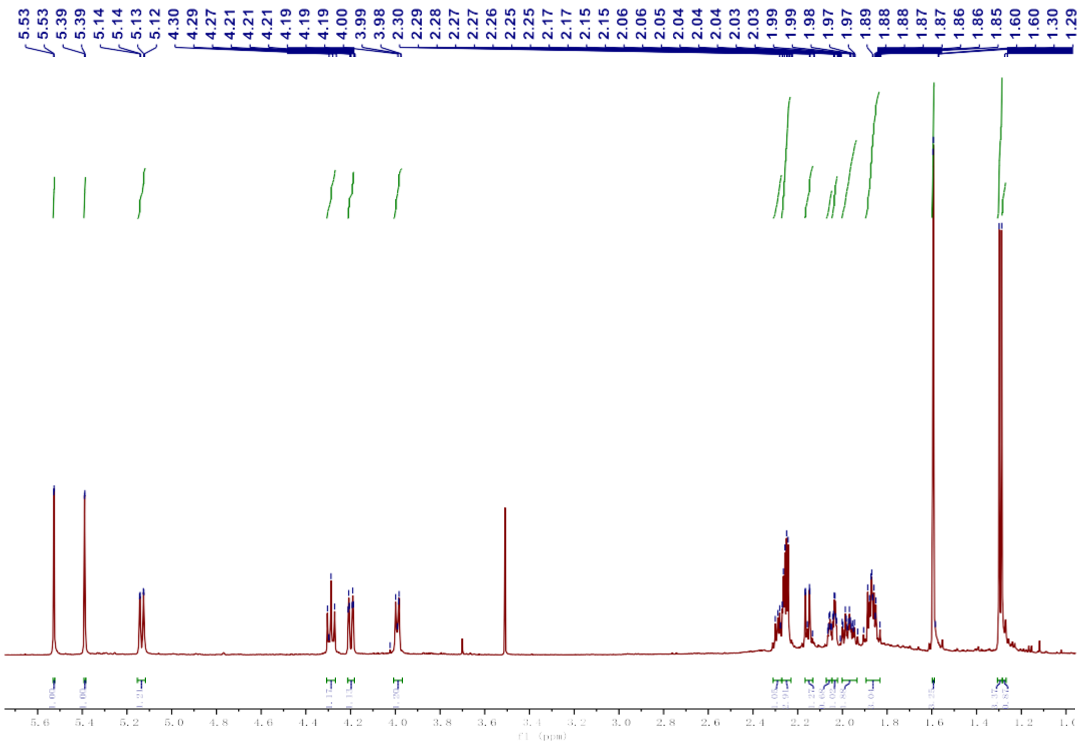

^{1}H NMR spectrum of **3** in $CDCl_3$

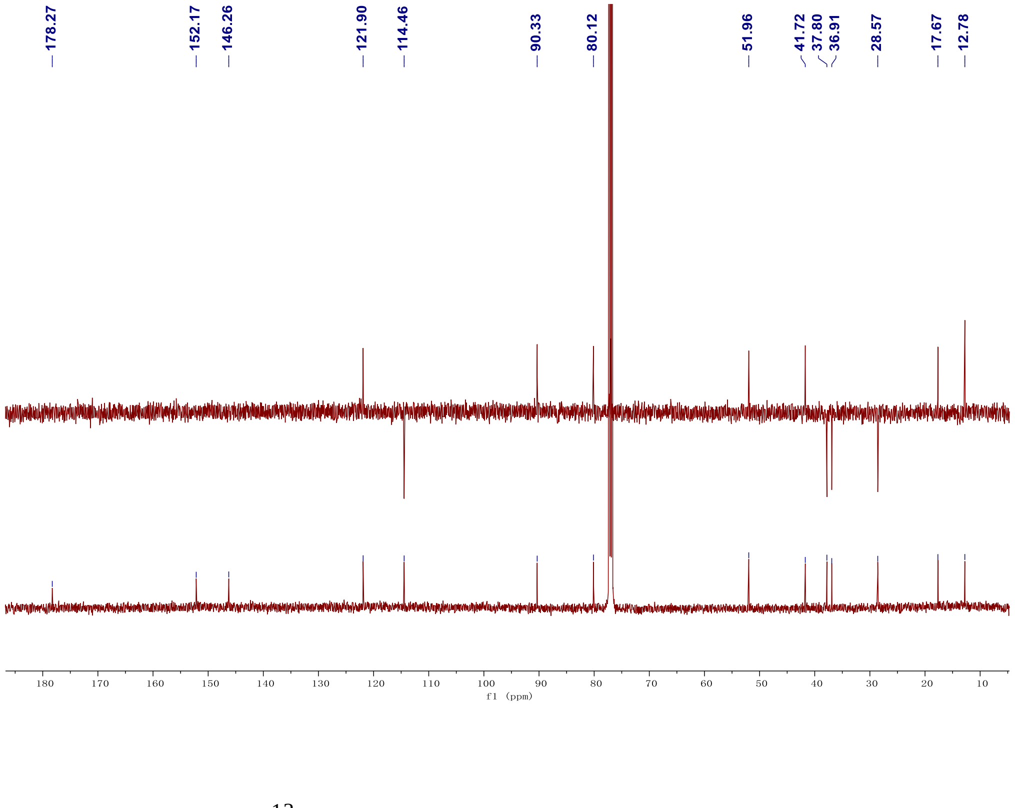

^{13}C NMR, DEPT spectrum of **3** in $CDCl_3$.

Figure S3. ^{1}H NMR, ^{13}C NMR, DEPT spectrum of **3** in $CDCl_3$.

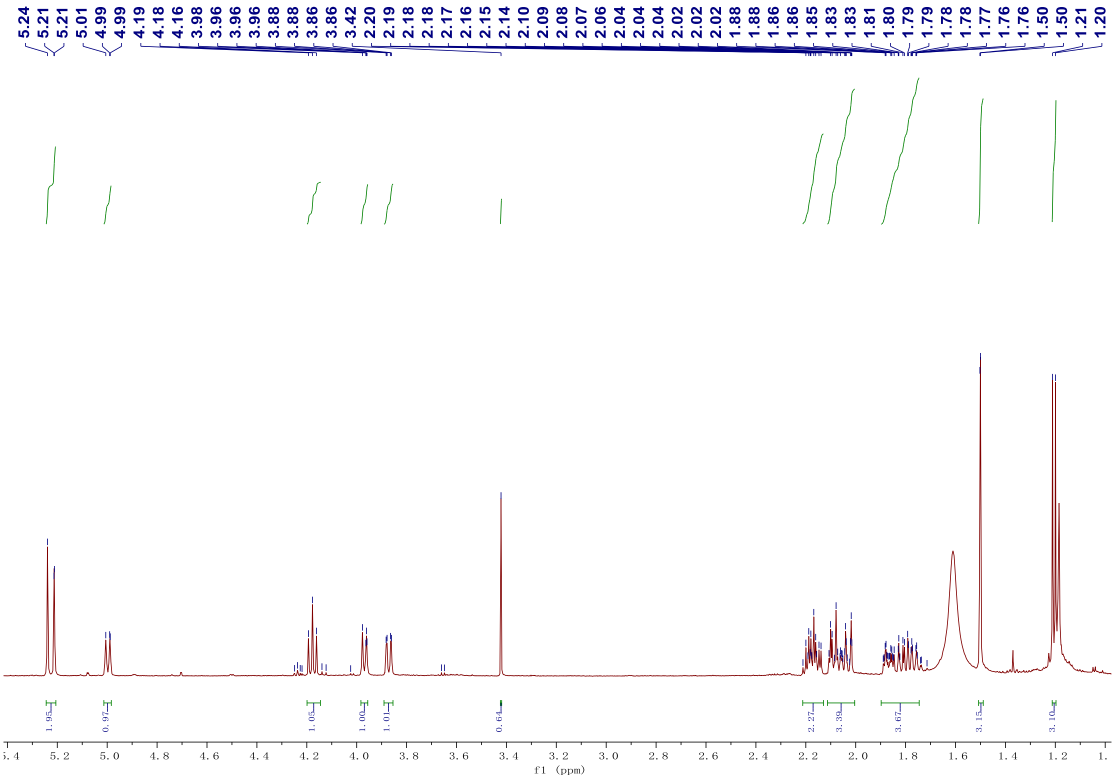

^{1}H NMR spectrum of **4** in $CDCl_3$

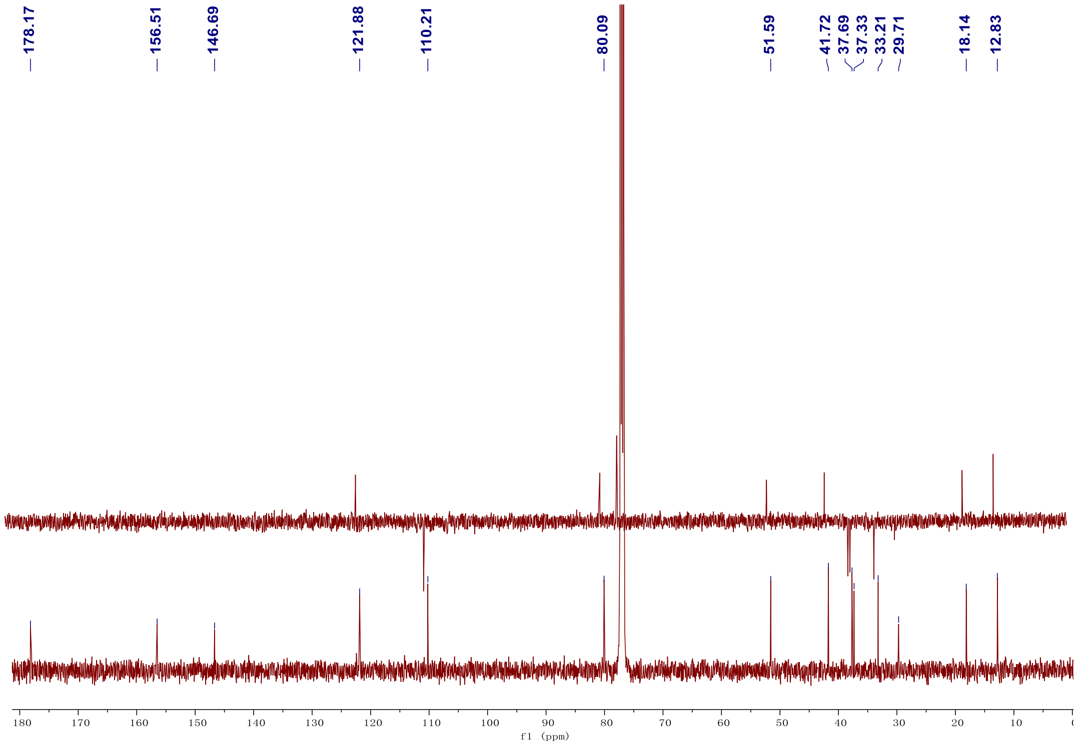

^{13}C NMR, DEPT spectrum of **4** in $CDCl_3$

Figure S4. ^{1}H NMR, ^{13}C NMR, DEPT spectrum of **4** in $CDCl_3$.

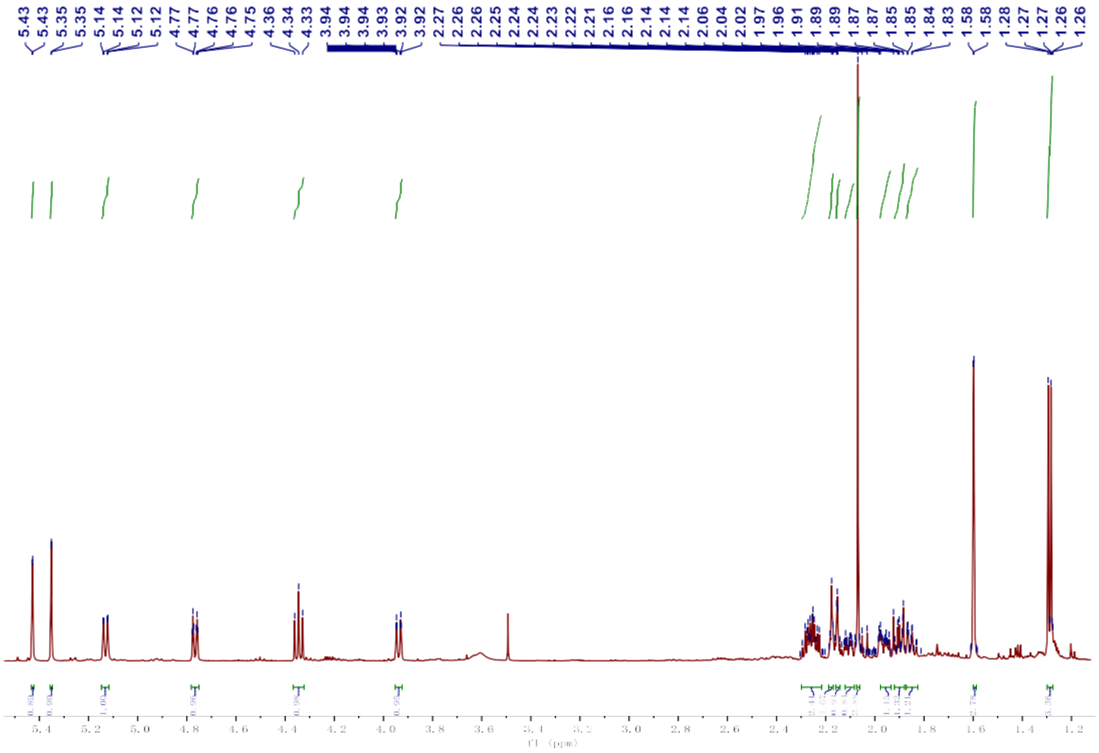

^{1}H NMR spectrum of **5** in $CDCl_3$

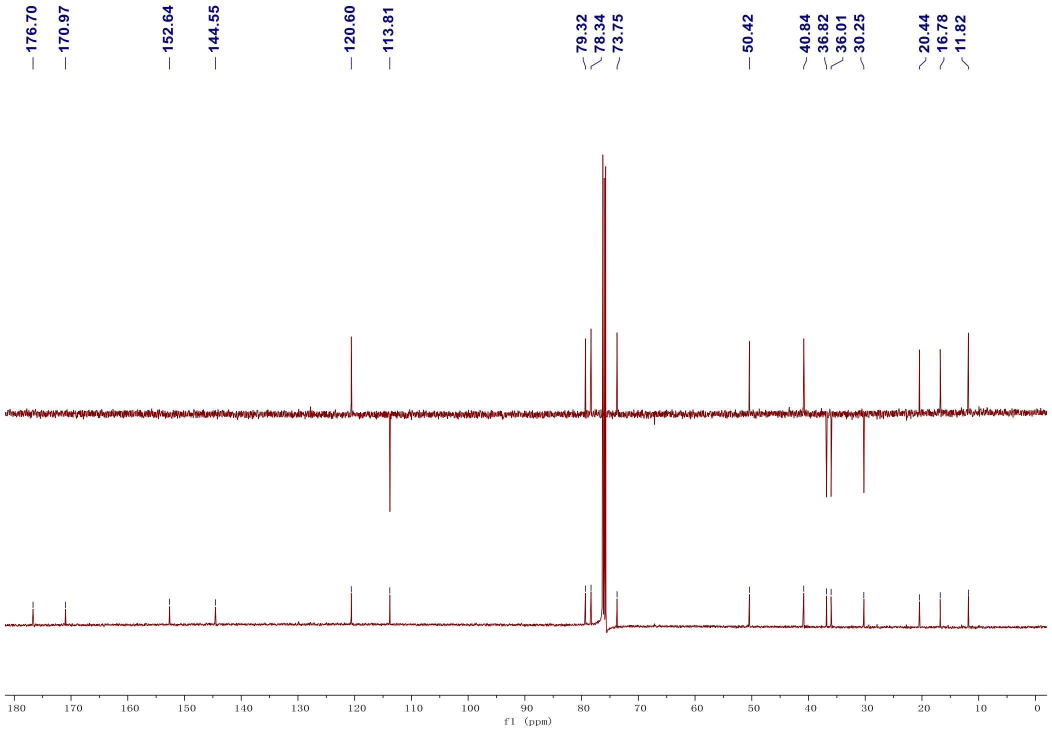

^{13}C NMR, DEPT spectrum of **5** in $CDCl_3$.

Figure S5. ^{1}H NMR, ^{13}C NMR, DEPT spectrum of **5** in $CDCl_3$.

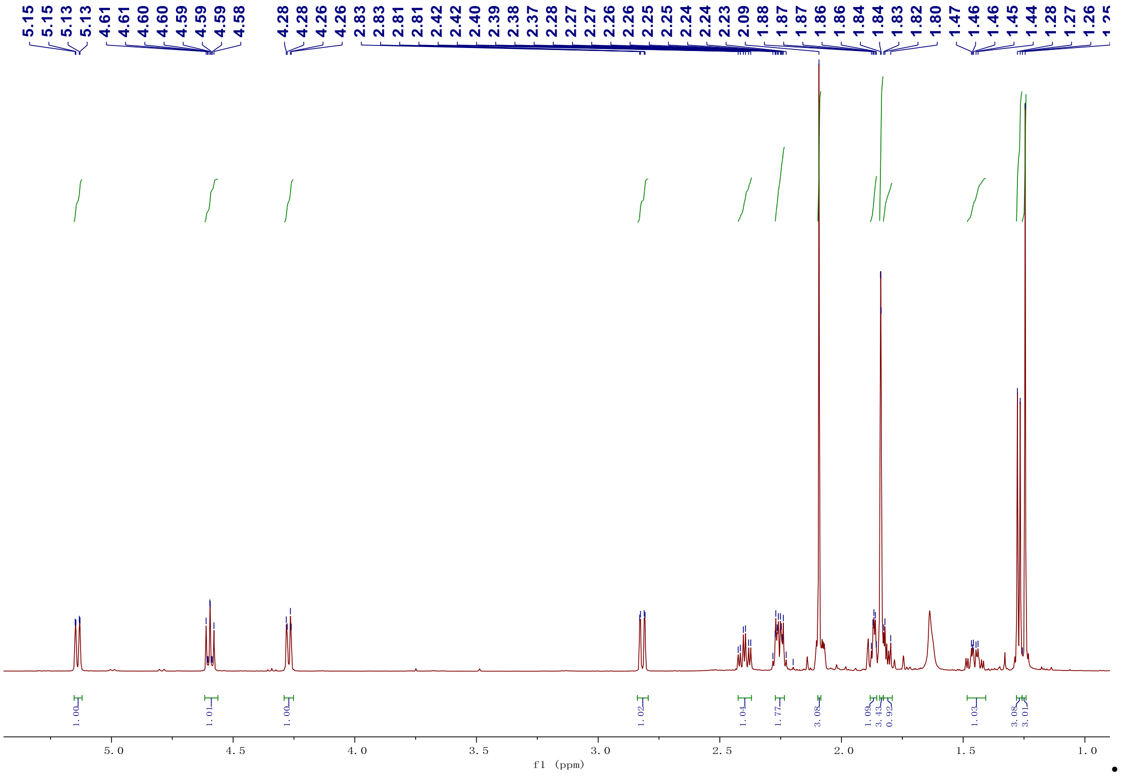

^{1}H NMR spectrum of **6** in $CDCl_3$

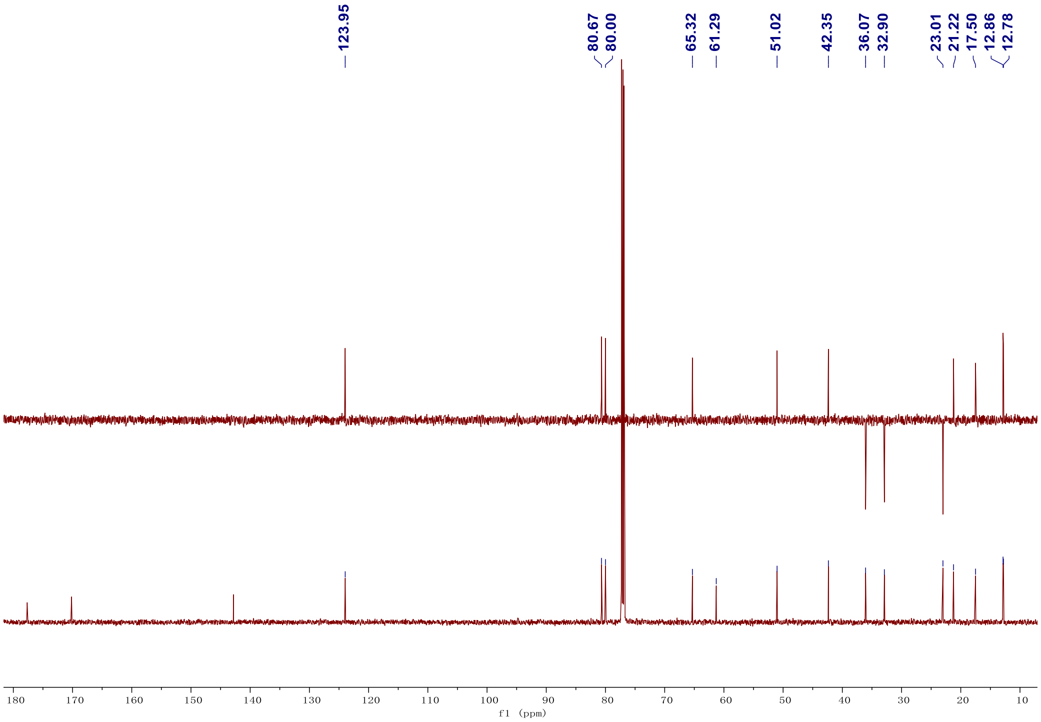

^{13}C NMR, DEPT spectrum of **6** in $CDCl_3$.

Figure S6. ^{1}H NMR, ^{13}C NMR, DEPT spectrum of **6** in CDCl.

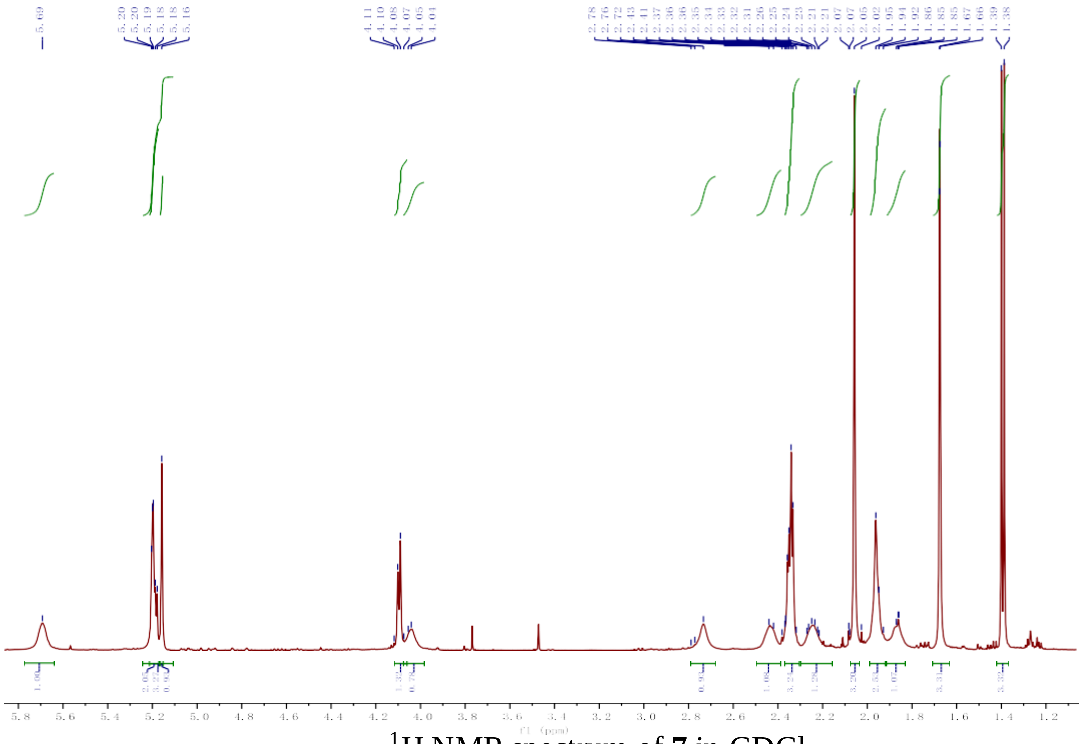

^{1}H NMR spectrum of **7** in $CDCl_3$

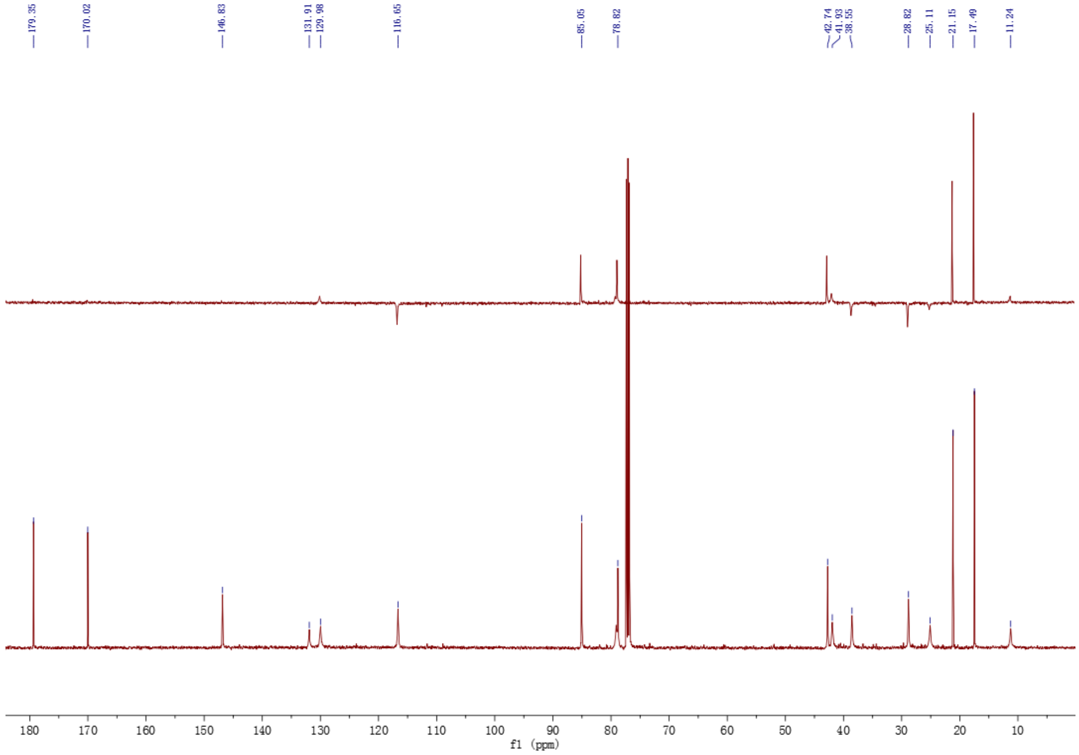

^{13}C NMR, DEPT spectrum of **7** in $CDCl_3$

Figure S7. ^{1}H NMR, ^{13}C NMR, DEPT spectrum of **7** in $CDCl_3$

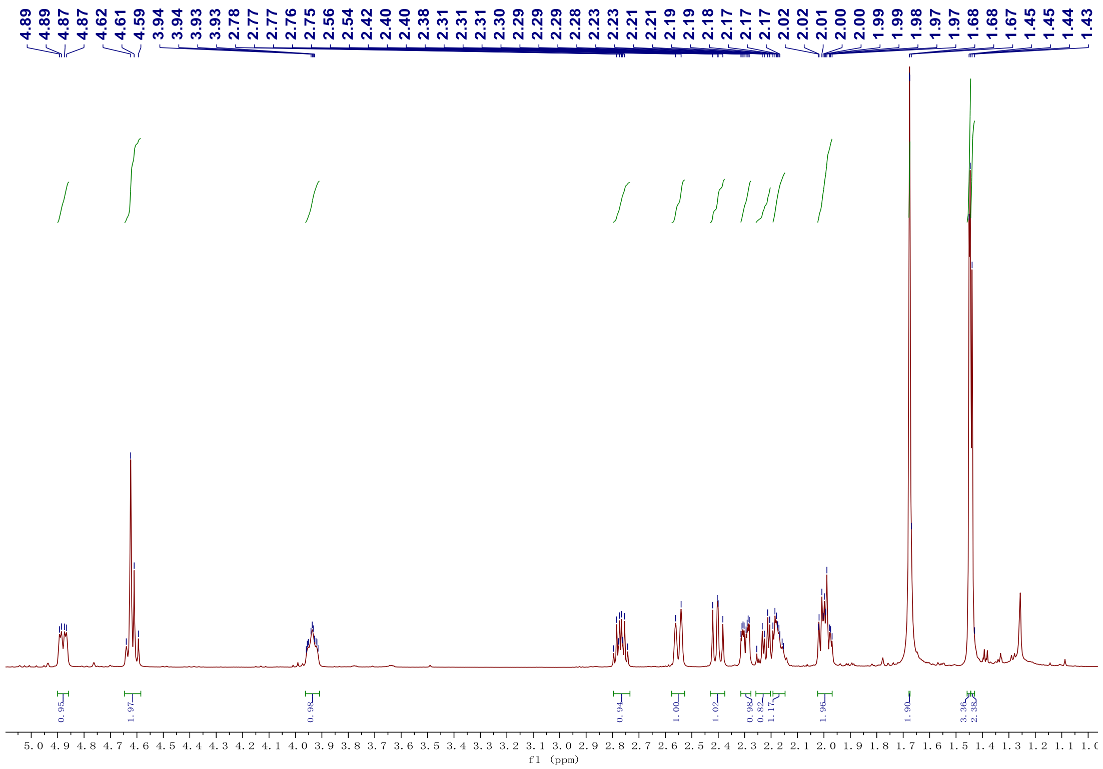

^{1}H NMR spectrum of **8** in $CDCl_3$

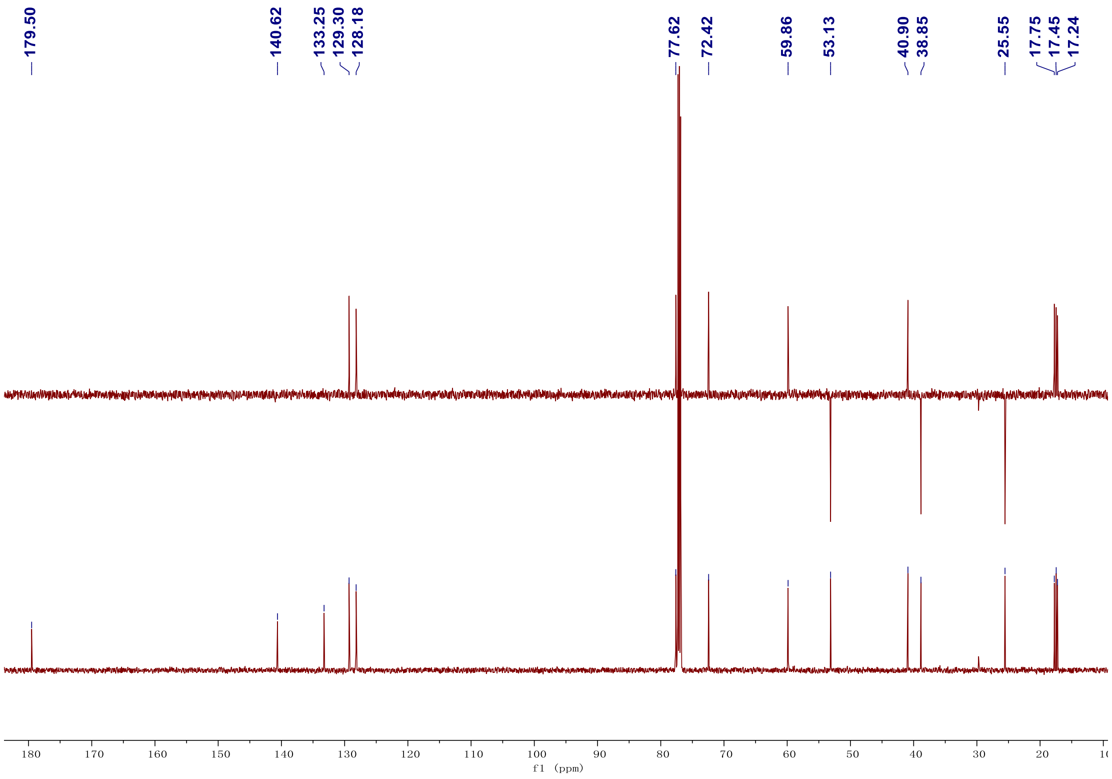

^{13}C NMR, DEPT spectrum of **8** in $CDCl_3$.

Figure S8. ^{1}H NMR, ^{13}C NMR, DEPT spectrum of **8** in $CDCl_3$.

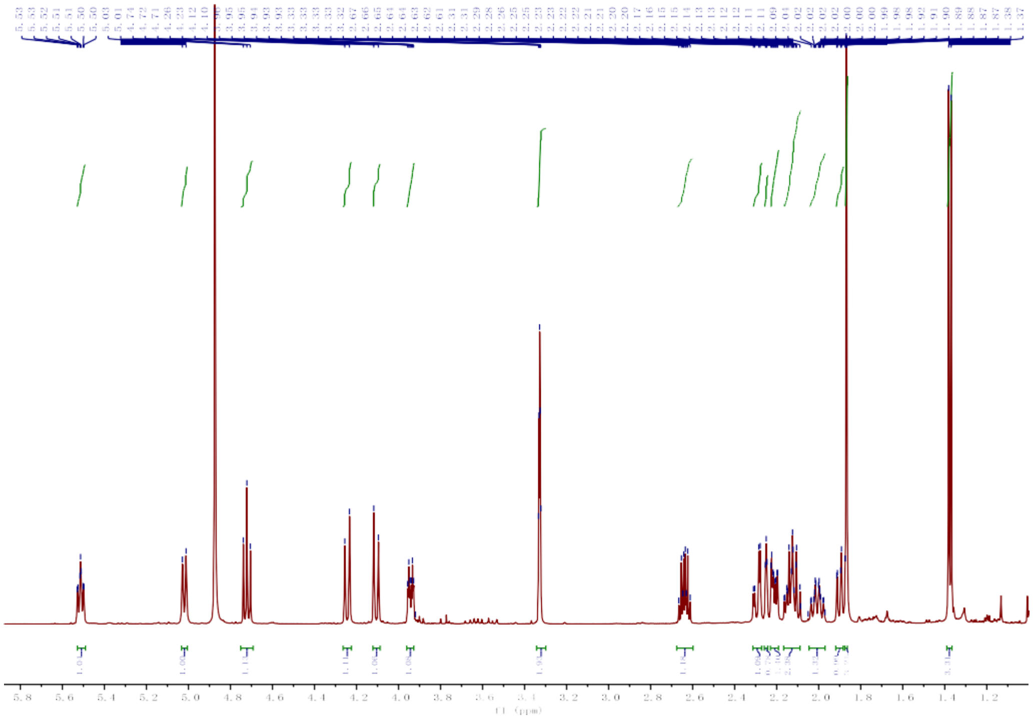

^{1}H NMR spectrum of **9** in CD_3OD

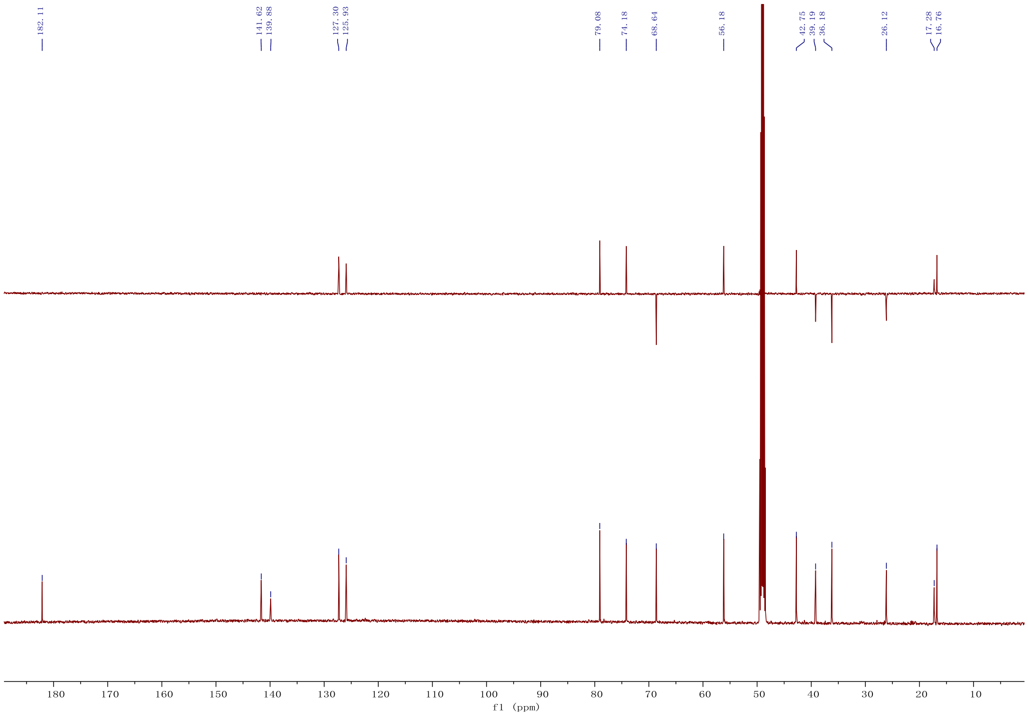

^{13}C NMR, DEPT spectrum of **9** in CD_3OD.

Figure S9. ^{1}H NMR, ^{13}C NMR, DEPT spectrum of **9** in CD_3OD.

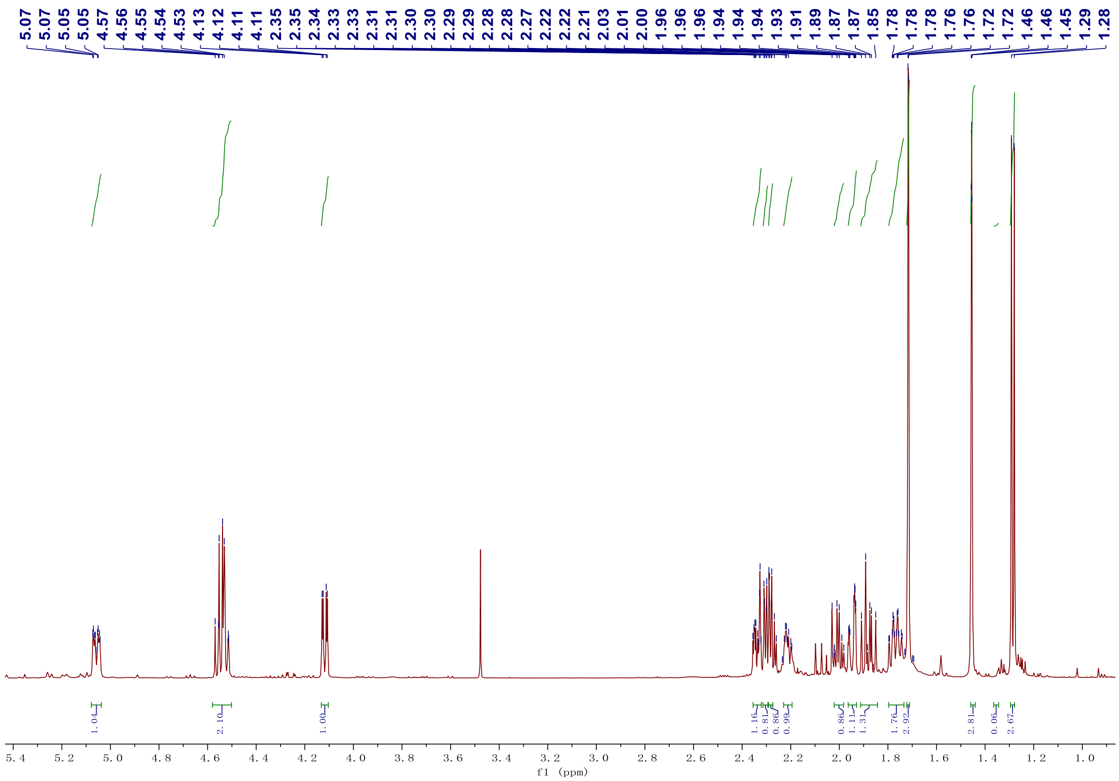

^{1}H NMR spectrum of compound **10** in $CDCl_3$.

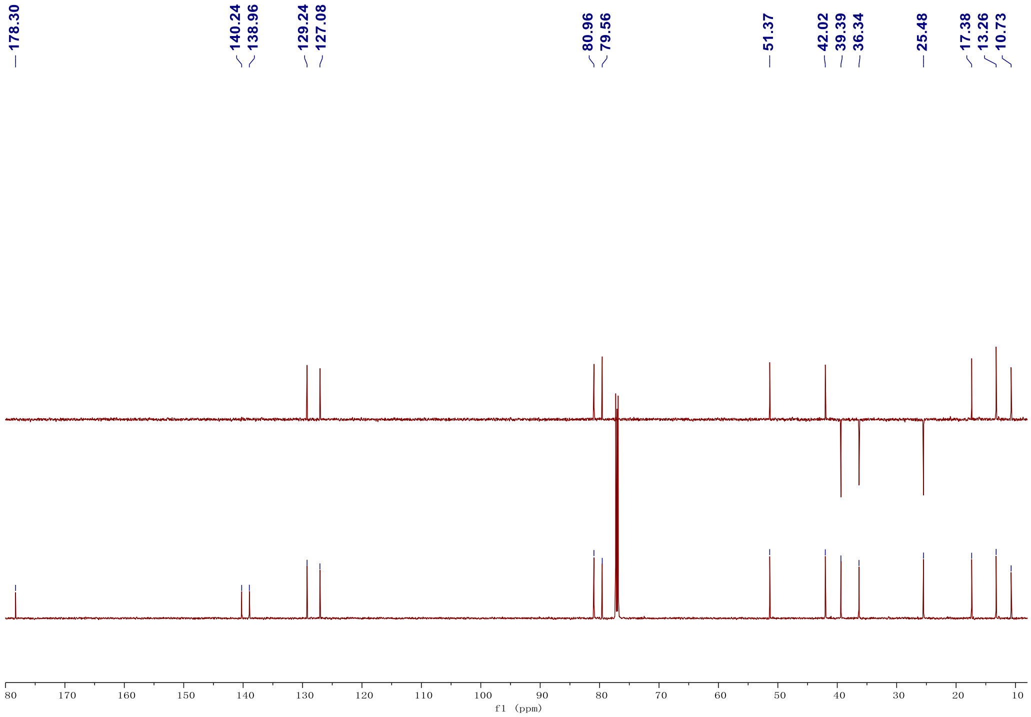

^{13}C NMR, DEPT spectrum of compound **10** in $CDCl_3$

Figure S10. ^{1}H NMR, ^{13}C NMR, DEPT spectrum of compound **10** in $CDCl_3$.

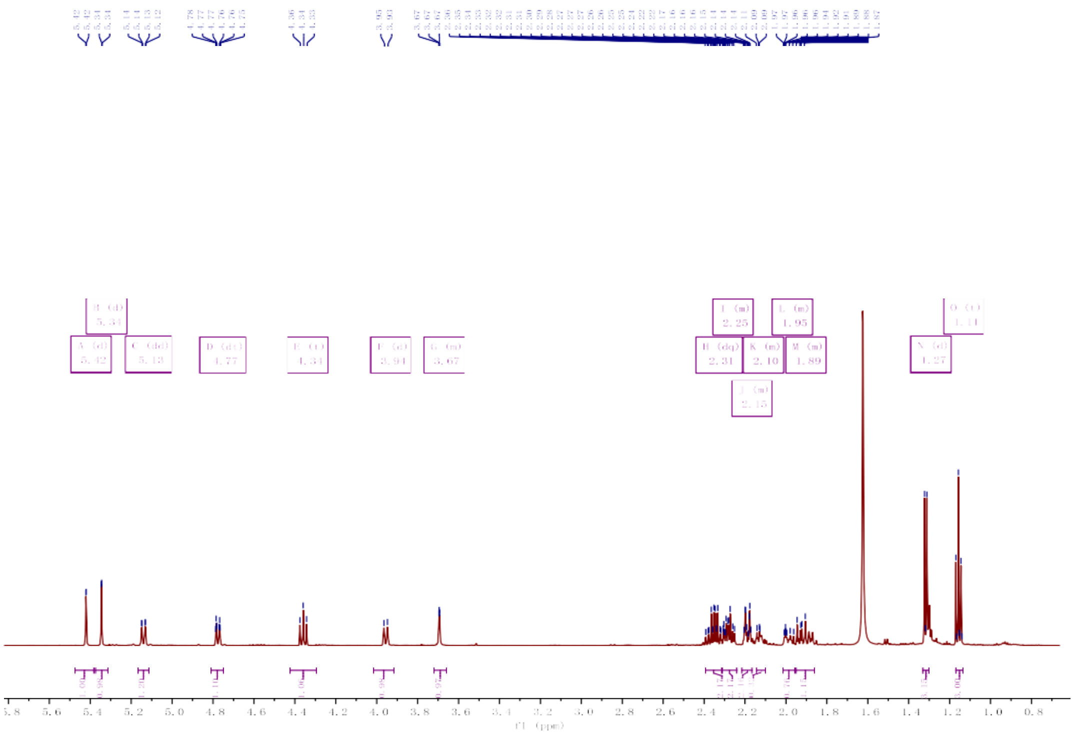

^{1}H NMR of Compound **11**

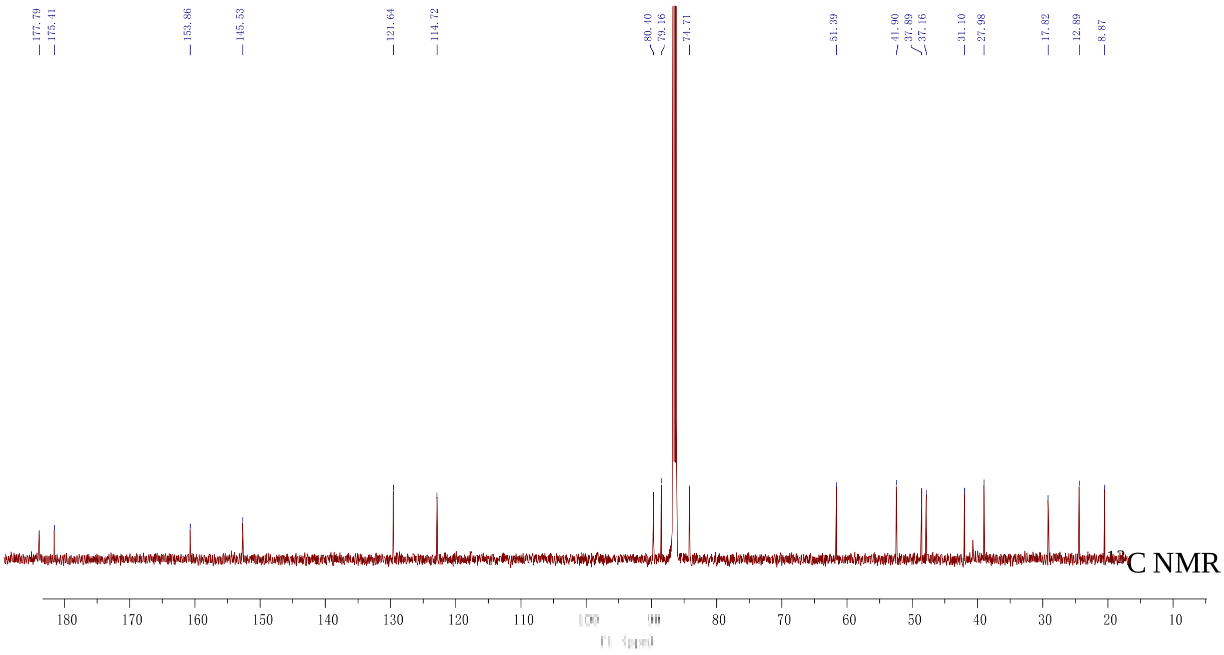

^{13}C NMR of Compound **11**

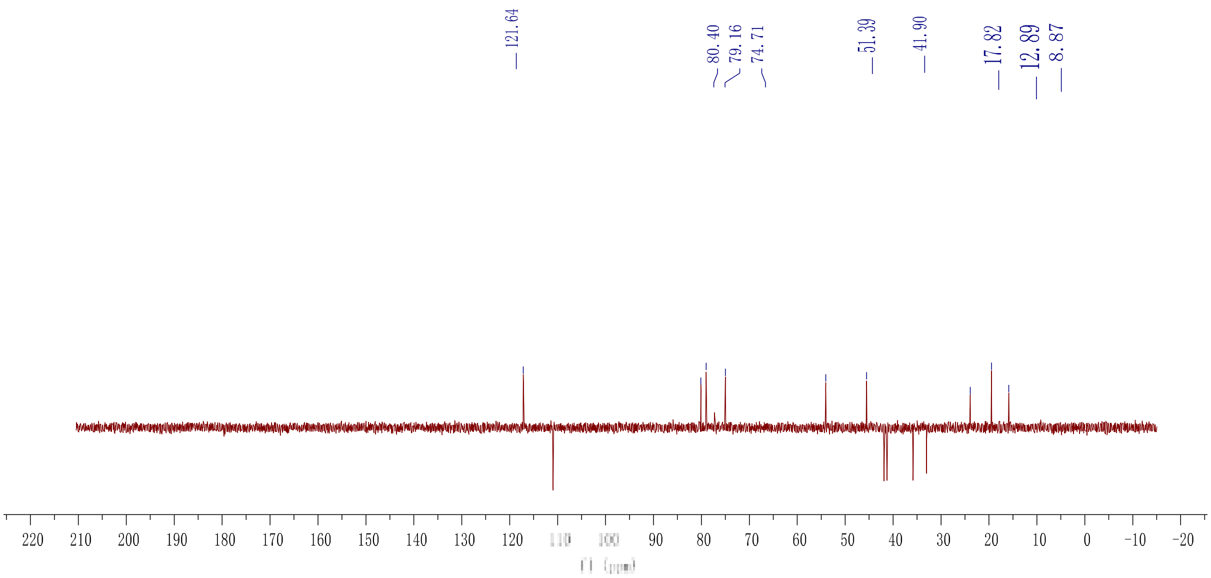

DEPT of Compound **11**

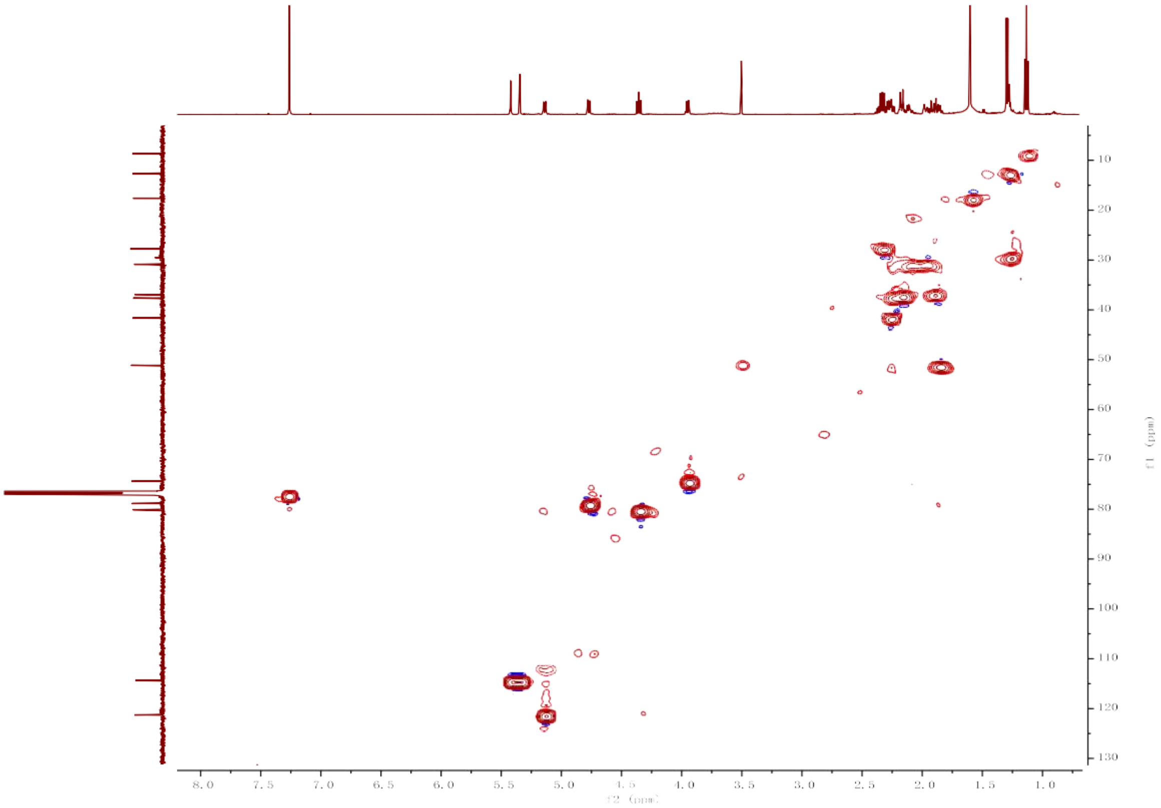

HSQC of Compound **11**

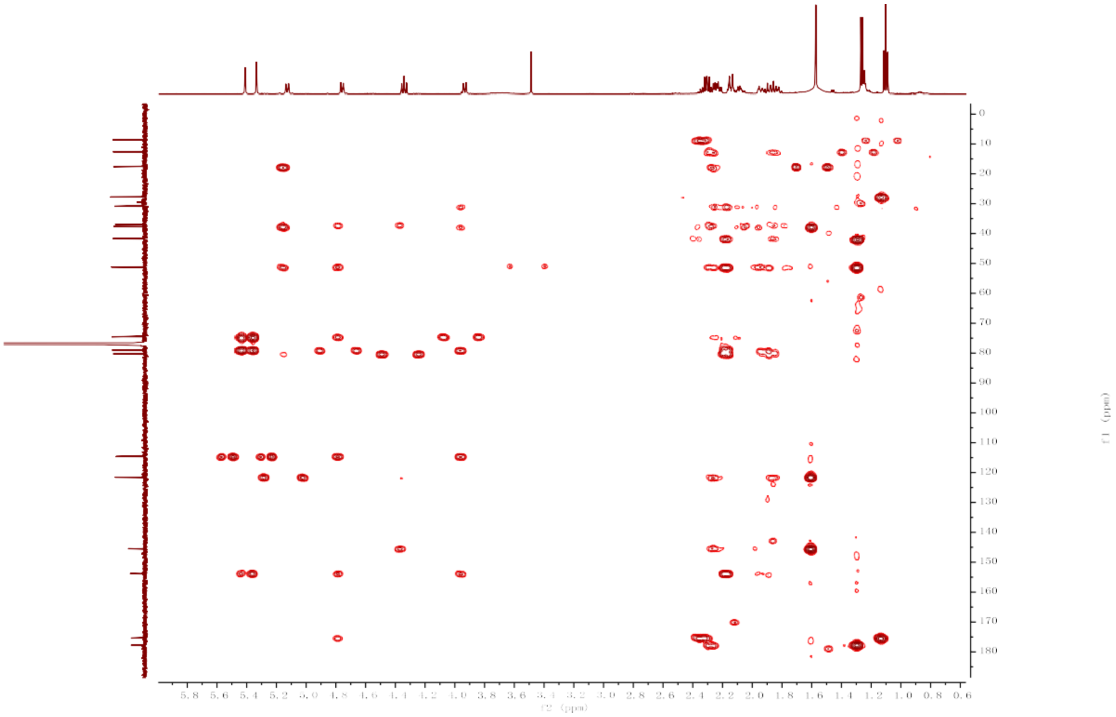

HMBC of Compound **11**

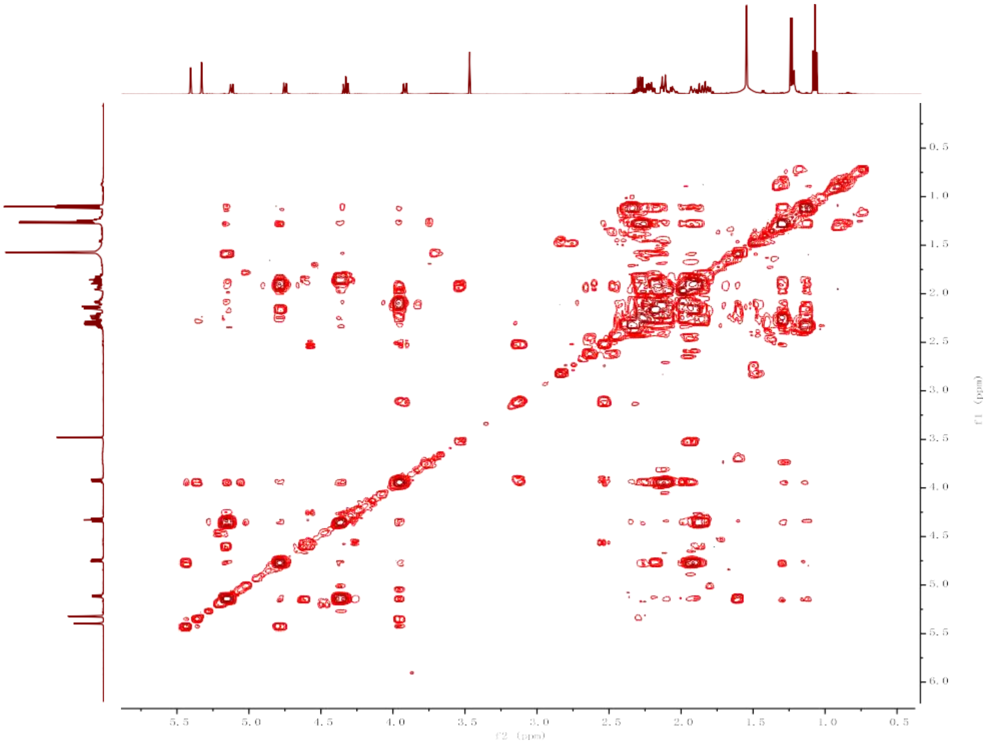

^1H-^1H COSY of Compound

Figure S11. ^1H NMR, ^{13}C NMR, DEPT, HSQC, HMBC and ^1H-^1H COSY spectra of **11** in $CDCl_3$

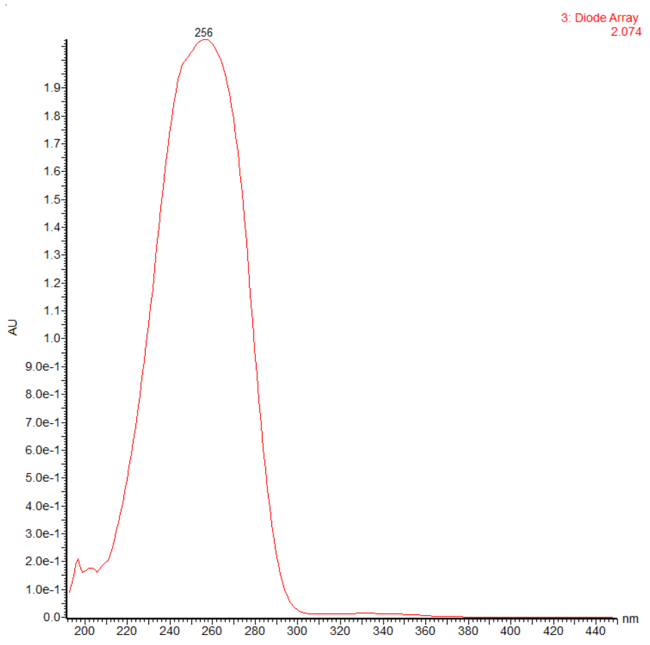

Figure S12. UV spectrum of **11** in MeOH.

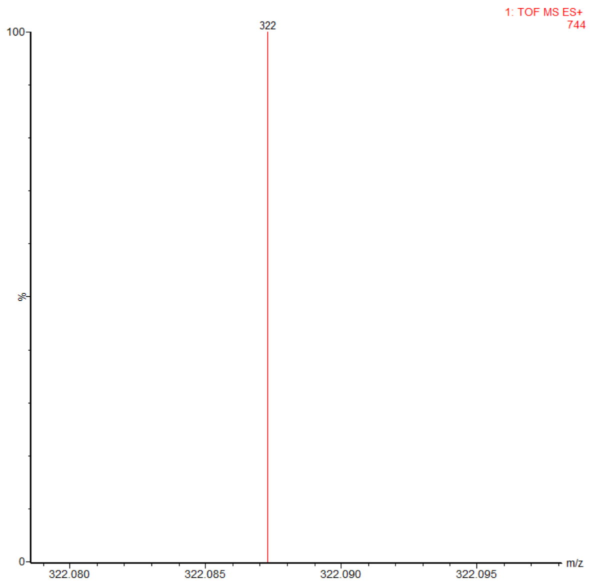

Figure S13. TOFMS spectral analysis of **11.**

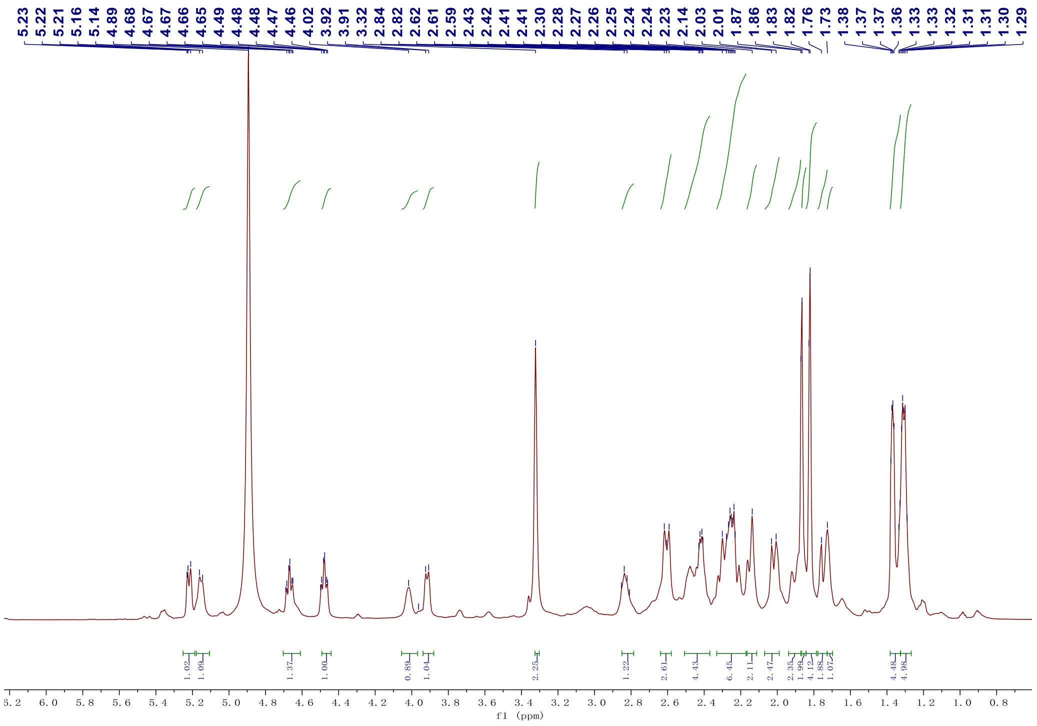

^{1}H NMR spectrum of **12** in $CDCl_3$.

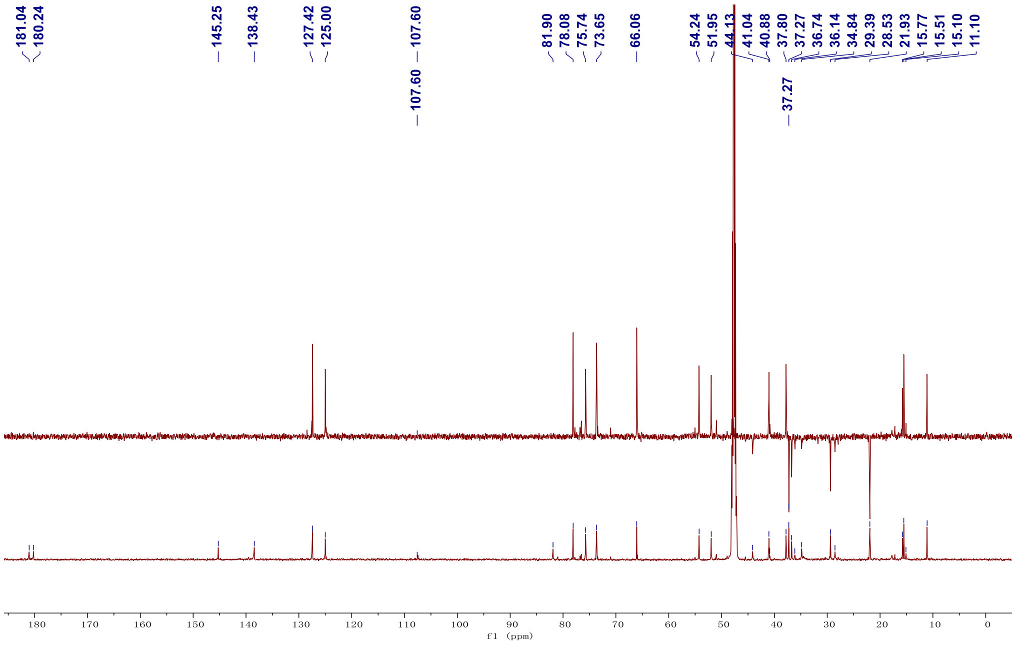

^{13}C NMR and DEPT spectrum of **12** in $CDCl_3$

Figure S14. ^{1}H NMR,^{13}C NMR, and DEPT spectrum of **12** in $CDCl_3$.

Supplementary data for *Artemisia marschalliana*

^{1}H NMR of Compound **1'**

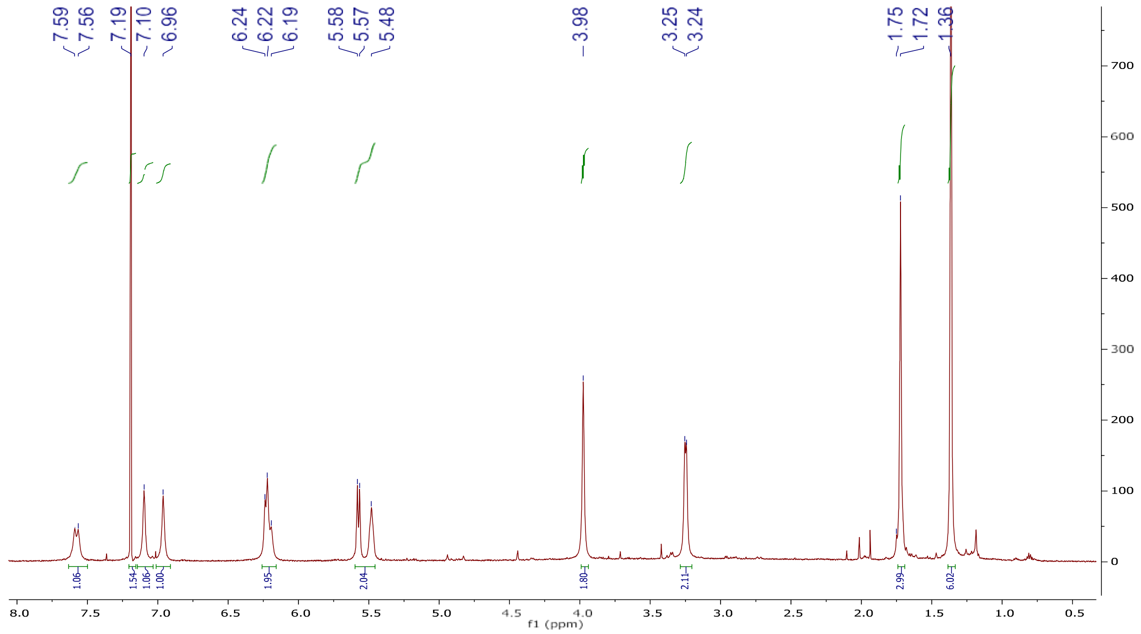

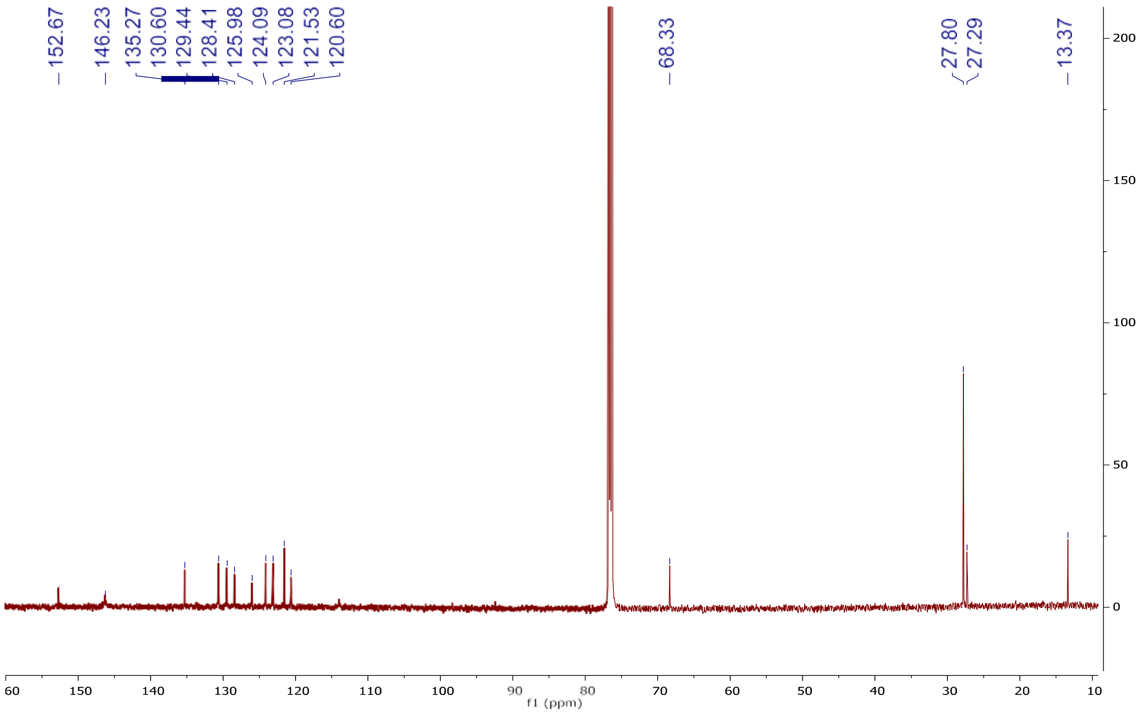

^{13}C NMR of Compound **1'**

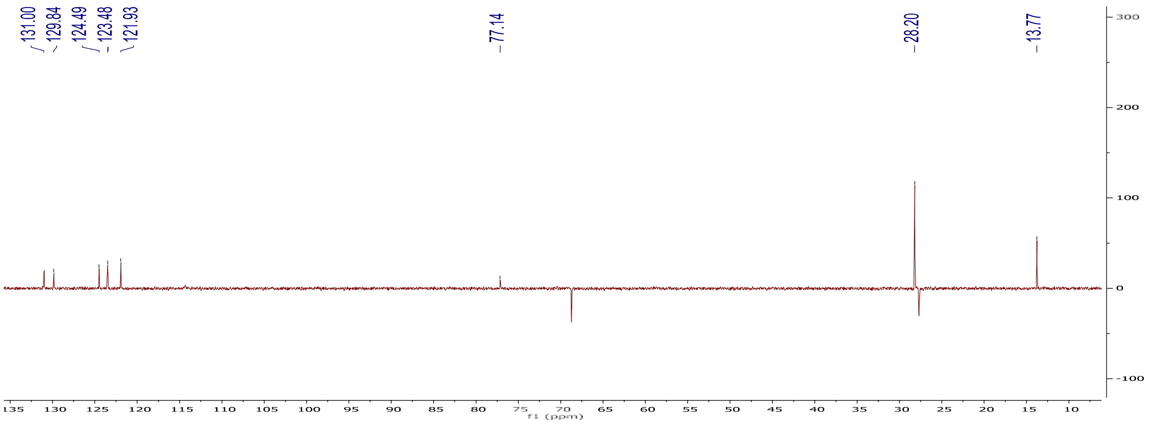

DEPT of Compound **1**'

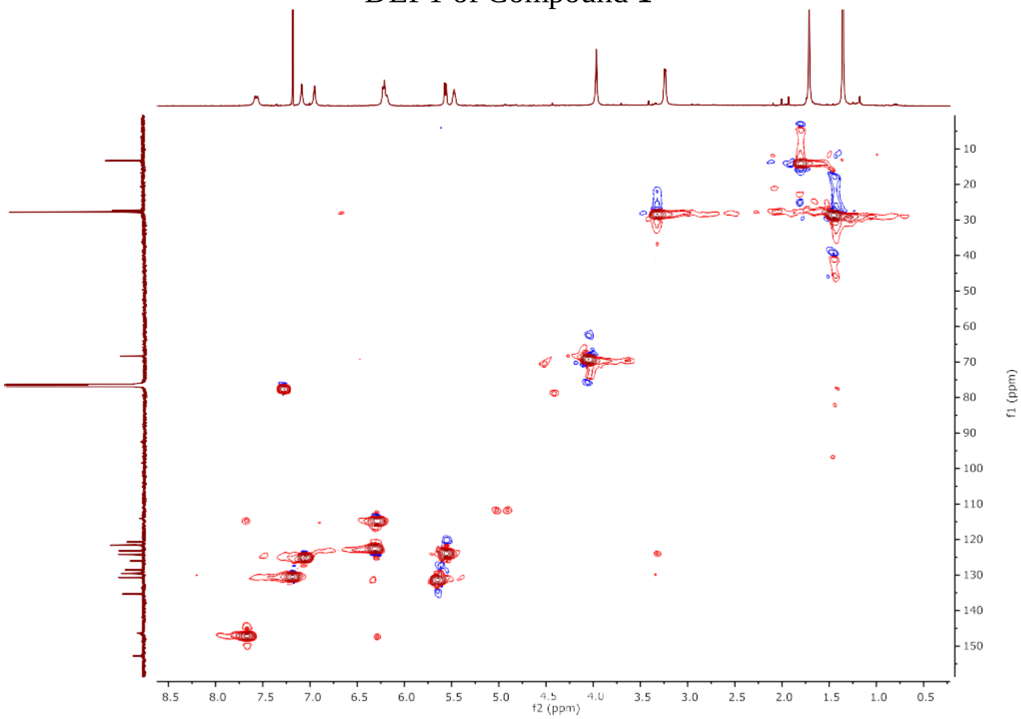

HSQC of Compound **1**'

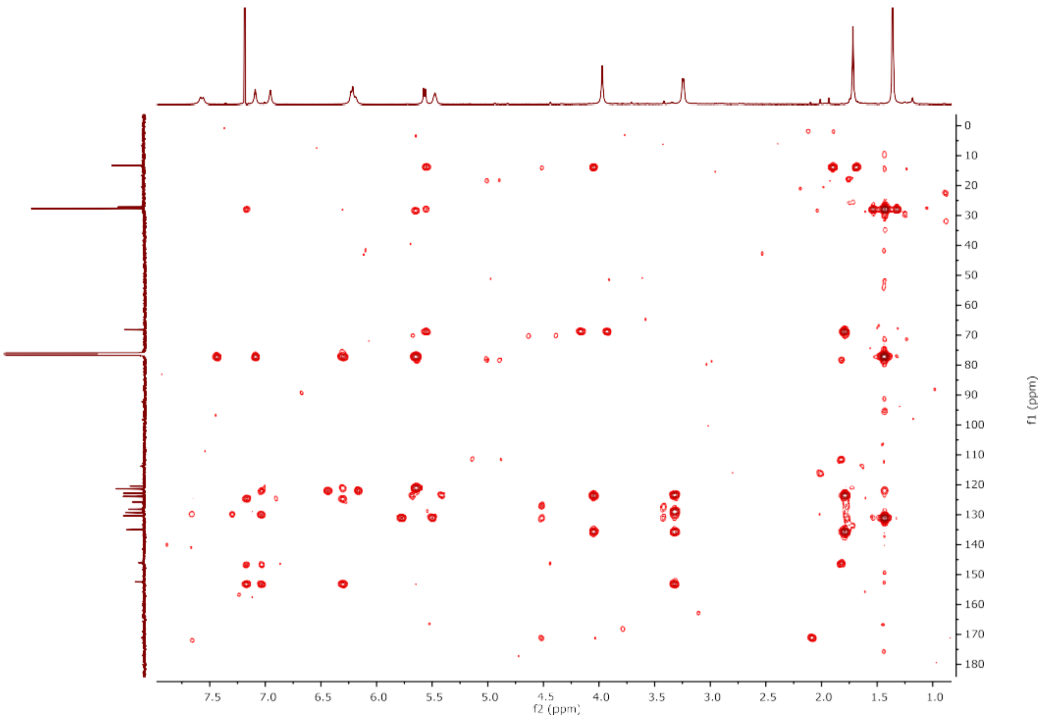

HMBC spectra of compound **1**’

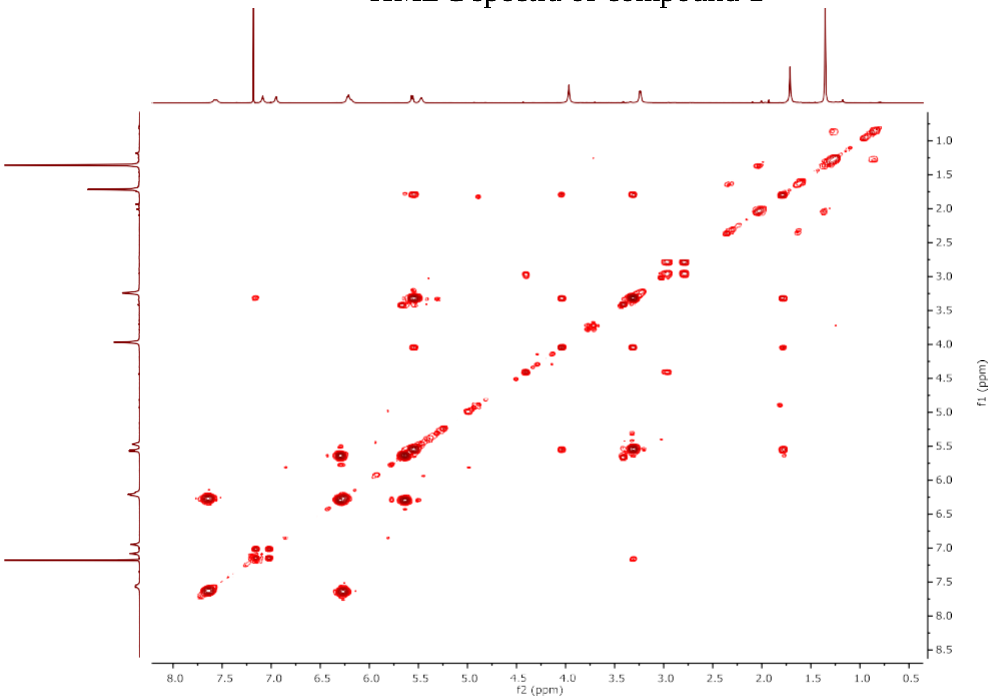

^{1}H-^{1}H COSY spectra of compound **1’**

Figure S15. ^{1}H NMR, ^{13}C NMR, DEPT, HSQC, HMBC and ^{1}H-^{1}H COSY spectra of **1’** in CDCl3

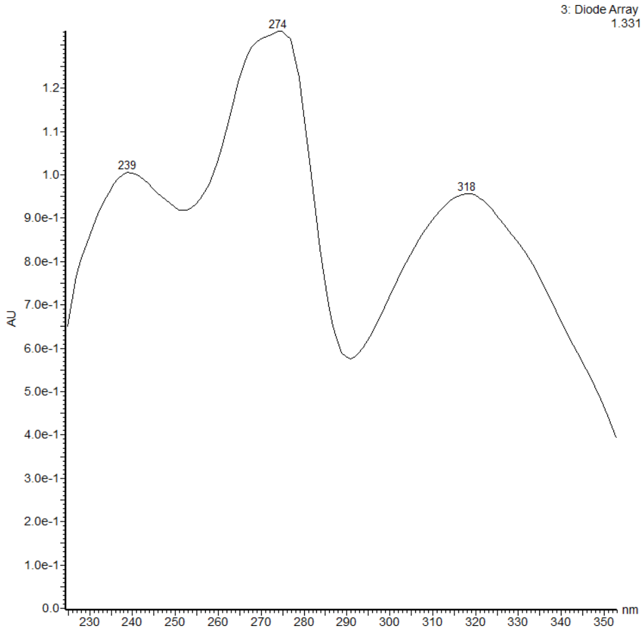

Figure S16. UV spectrum of Compound **1’** in MeOH.

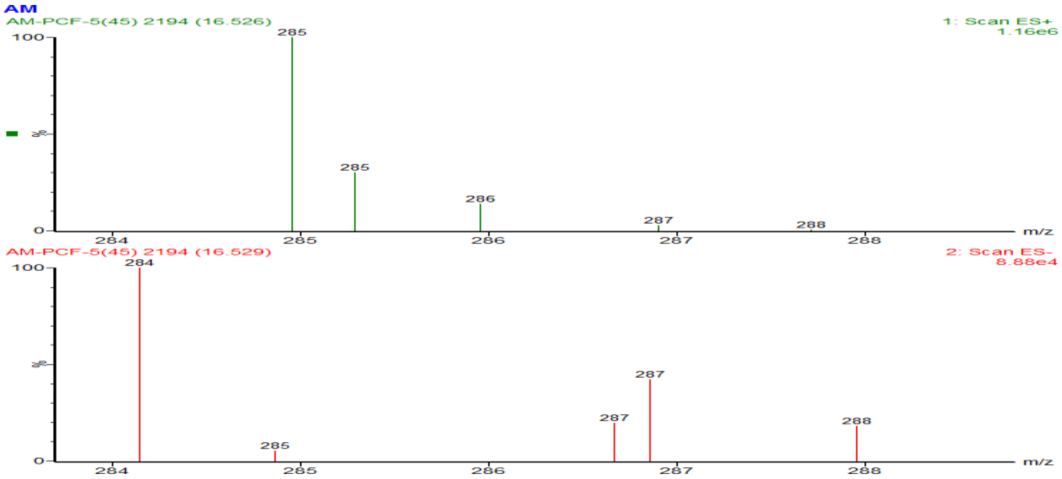

Figure S17. ESI-MS spectrum of Compound **1’**.

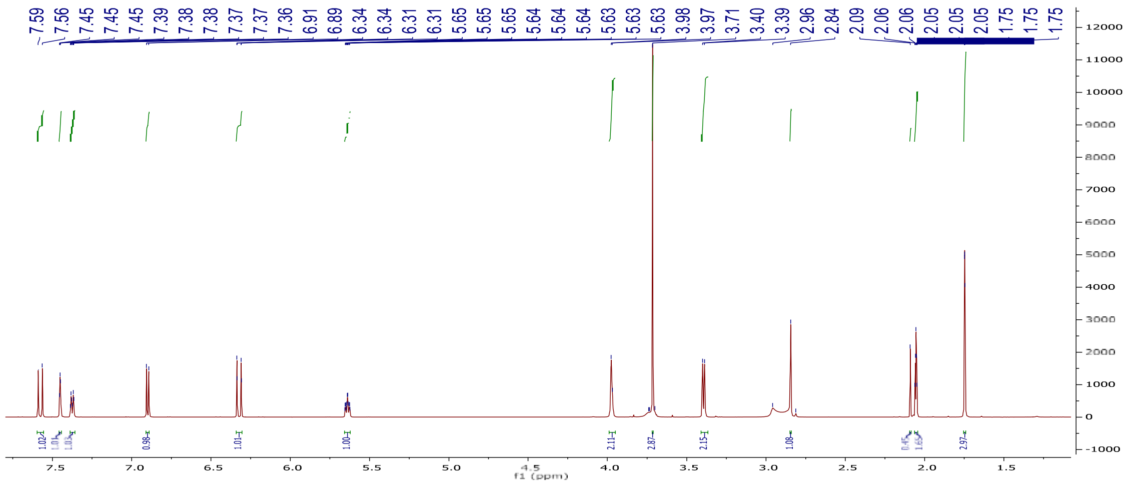

^{1}H NMR spectrum of **2'** in Acetone d-6

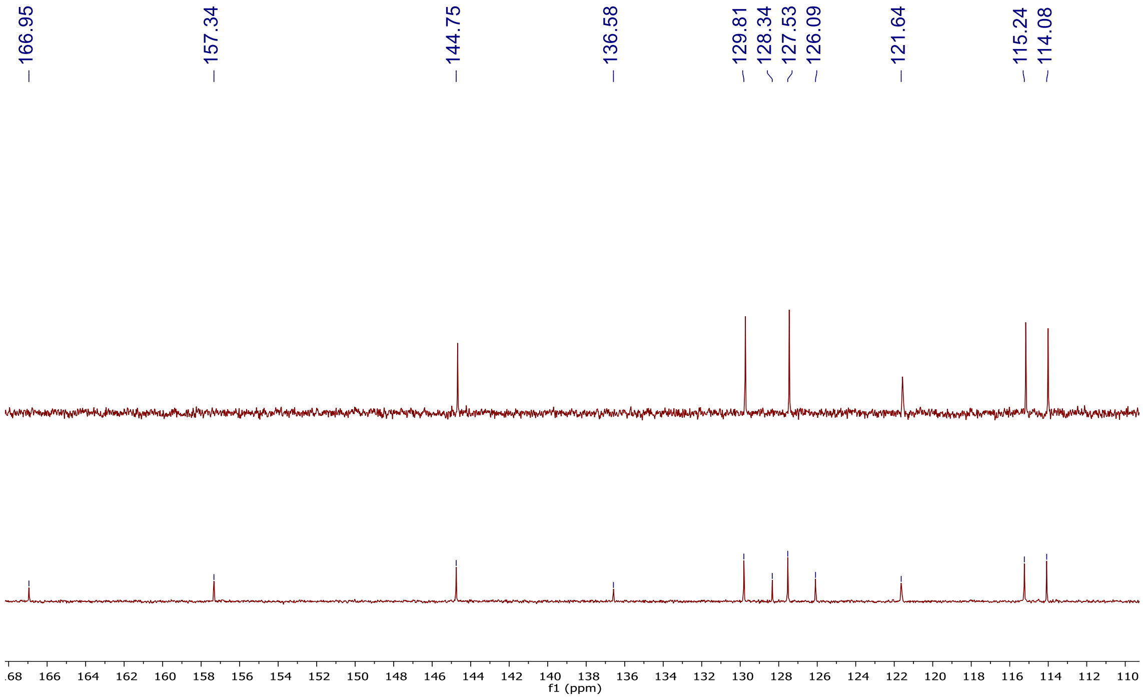

^{13}C NMR, DEPT spectrum of **2'** in Acetone d-6.

Figure S18. ^{1}H NMR, ^{13}C NMR, DEPT spectrum of **2'** in Acetone d-6.

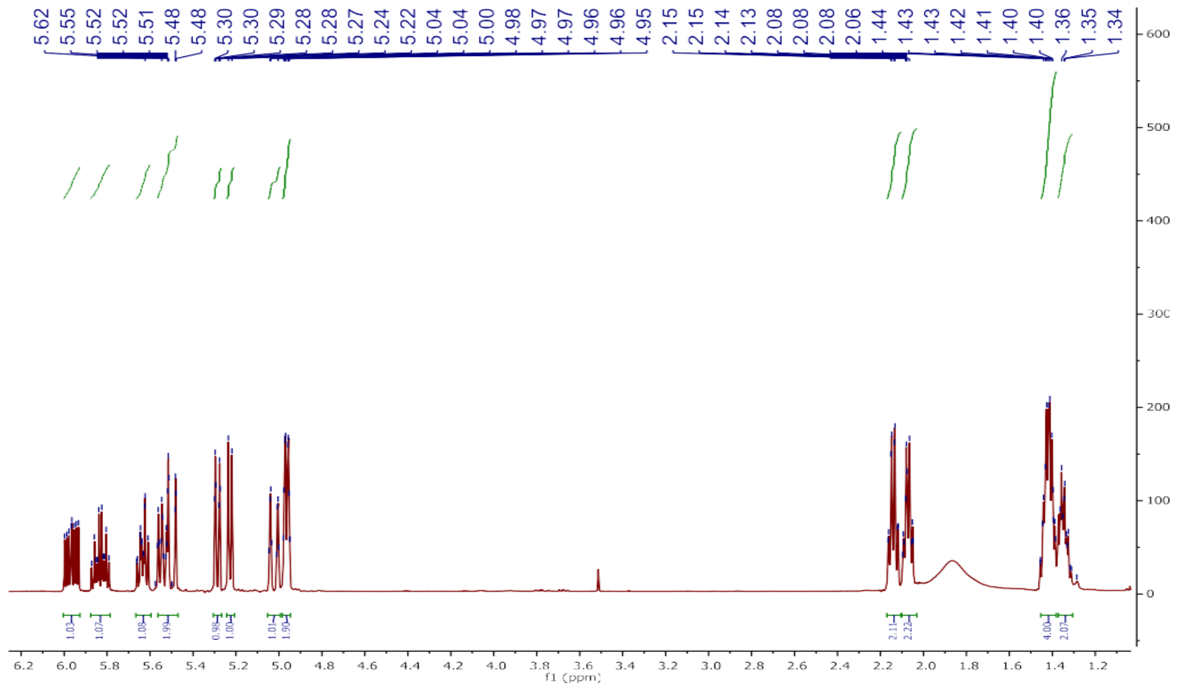

^{1}H NMR spectrum of **3’** in $CDCl_3$

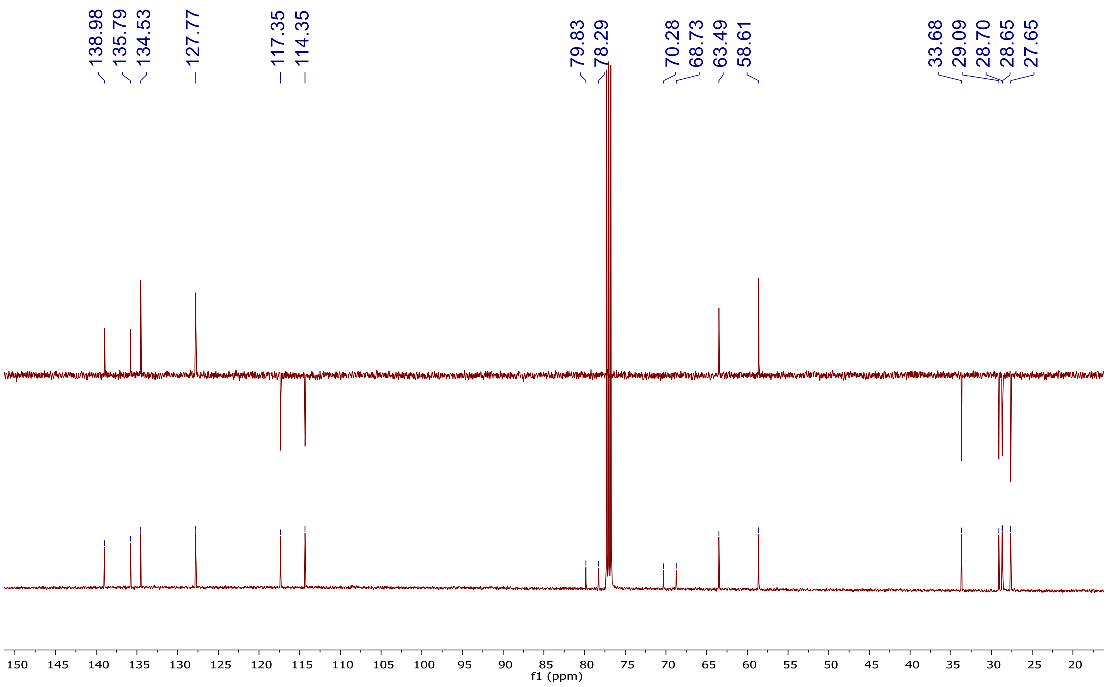

^{13}C NMR and DEPT spectrum of **3’** in $CDCl_3$.

Figure S19. ^{1}H NMR, ^{13}C NMR and DEPT spectrum of **3’** in $CDCl_3$

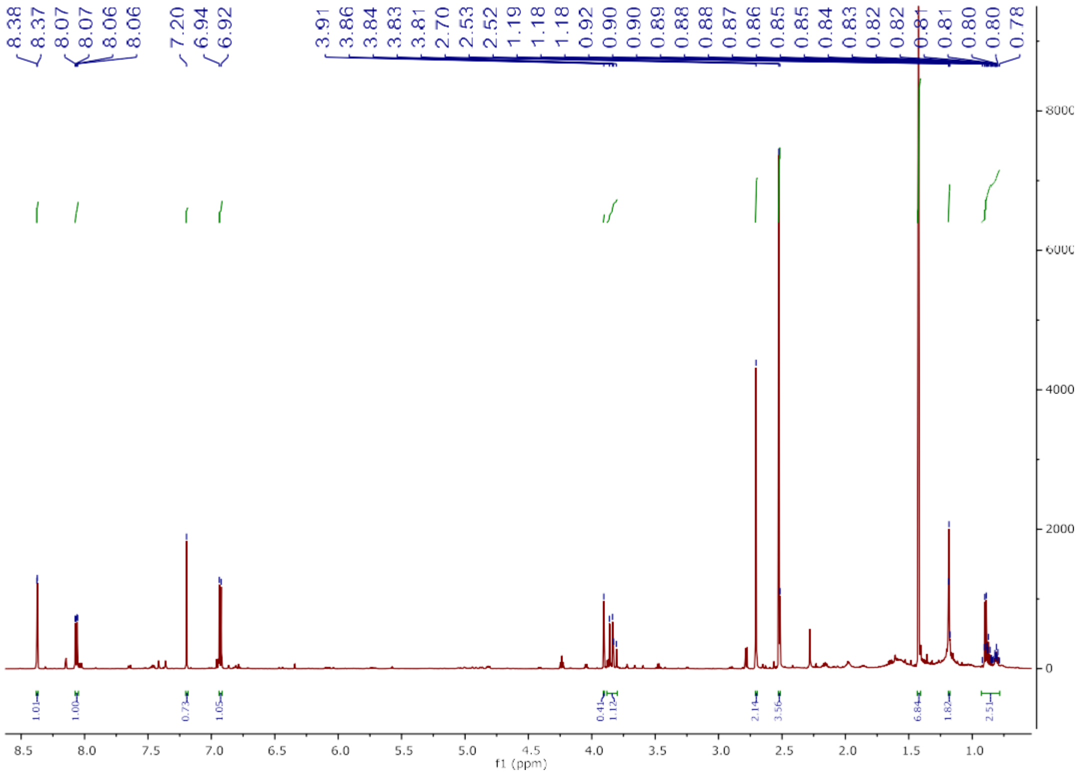

^{1}H NMR spectrum of **4’** in $CDCl_3$

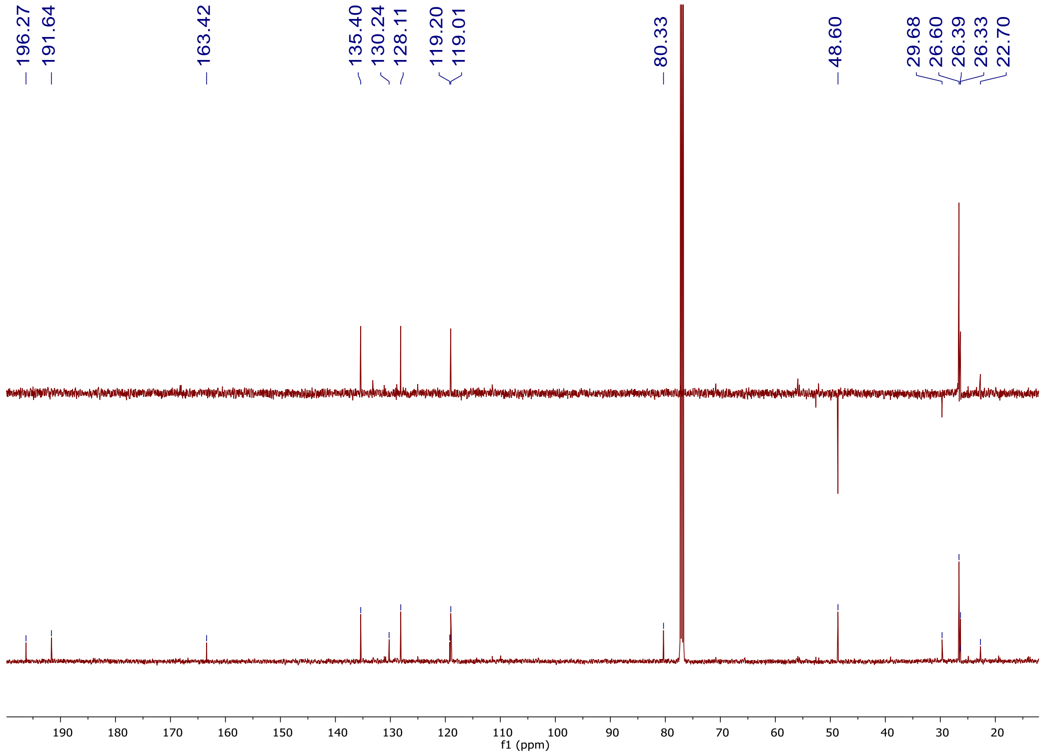

^{13}C NMR, DEPT spectrum of **4’** in $CDCl_3$

Figure S20. ^{1}H NMR, ^{13}C NMR, DEPT spectrum of **4’** in $CDCl_3$

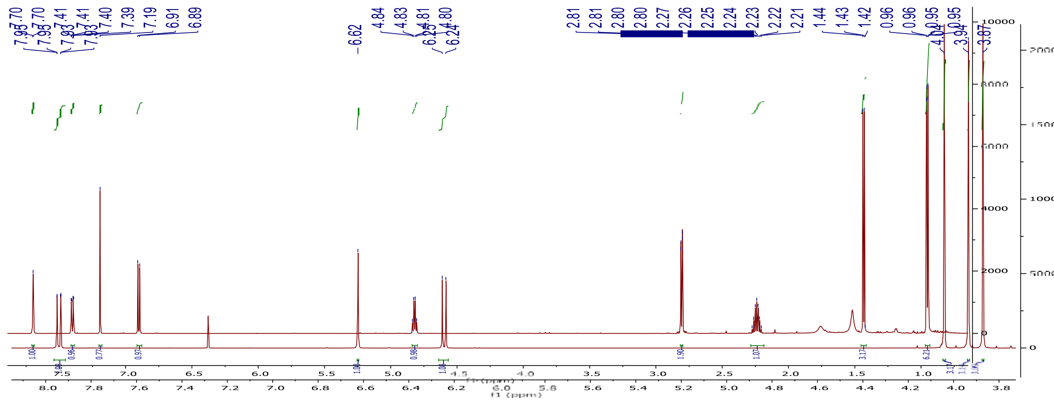

^{1}H NMR spectrum of **5’** in $CDCl_3$

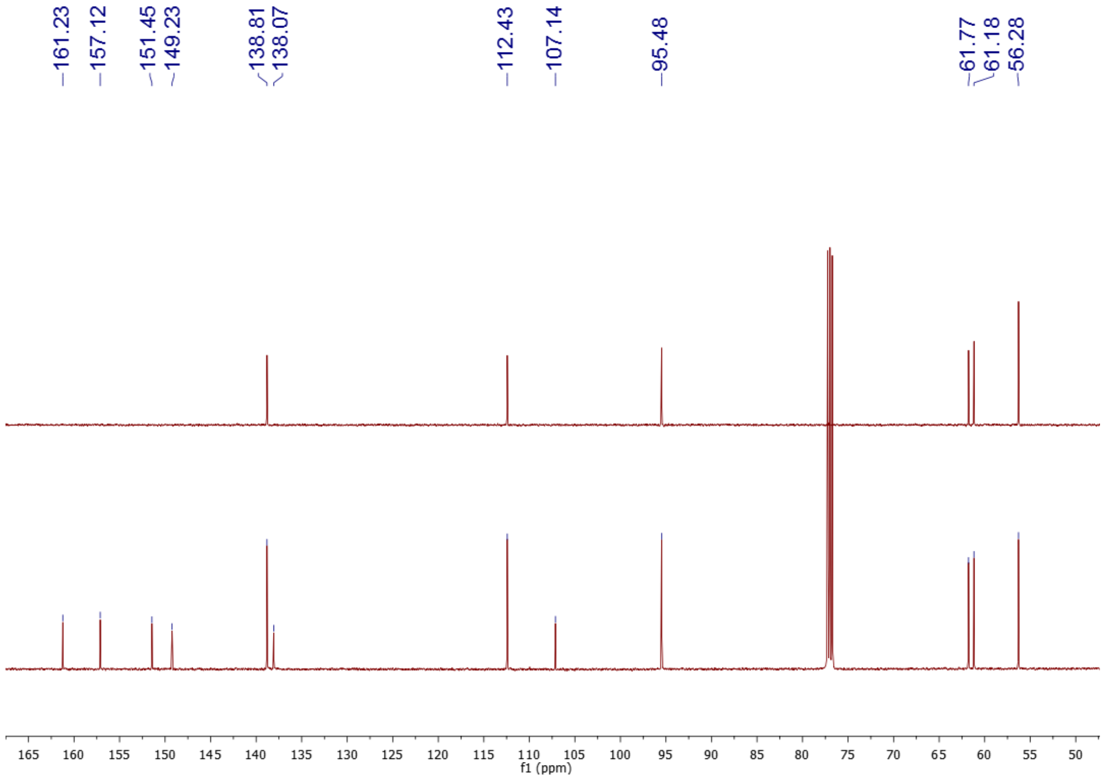

^{13}C NMR, DEPT spectrum of **5’** in $CDCl_3$.

Figure S21. ^{1}H NMR, ^{13}C NMR, DEPT spectrum of **5’** in $CDCl_3$.

^{1}H NMR spectrum of **6’** in $CDCl_3$

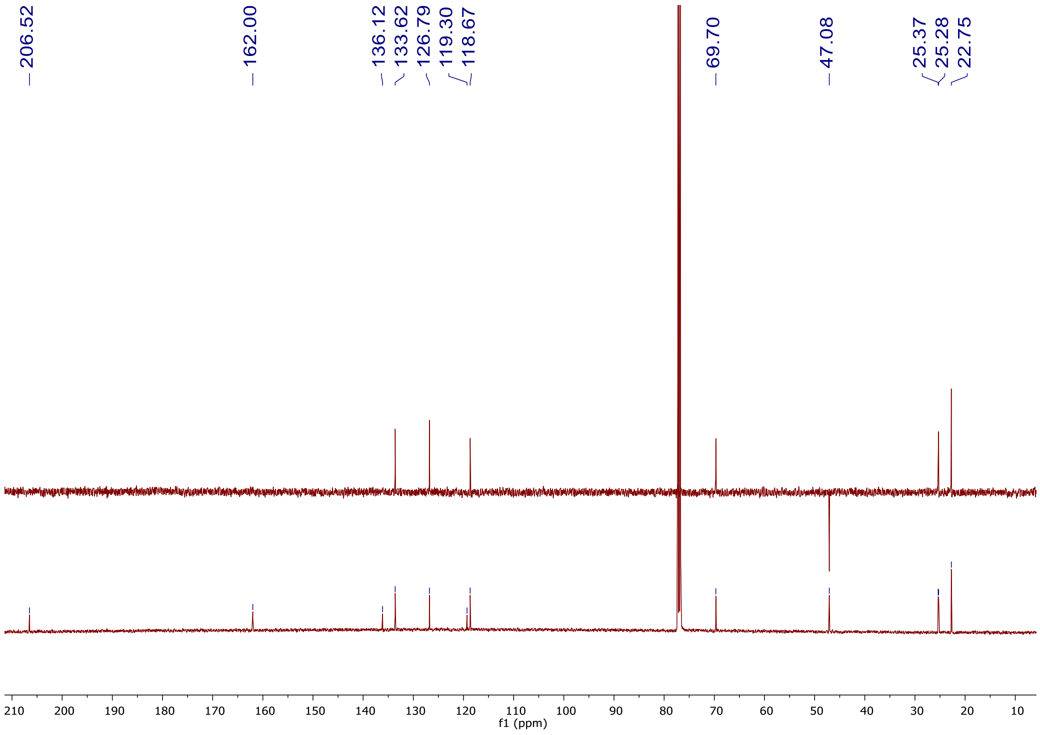

^{13}C NMR, DEPT spectrum of **6'** in $CDCl_3$

Figure S22. ^{1}H NMR, ,^{13}C NMR, DEPT spectrum of **6'** in $CDCl_3$.

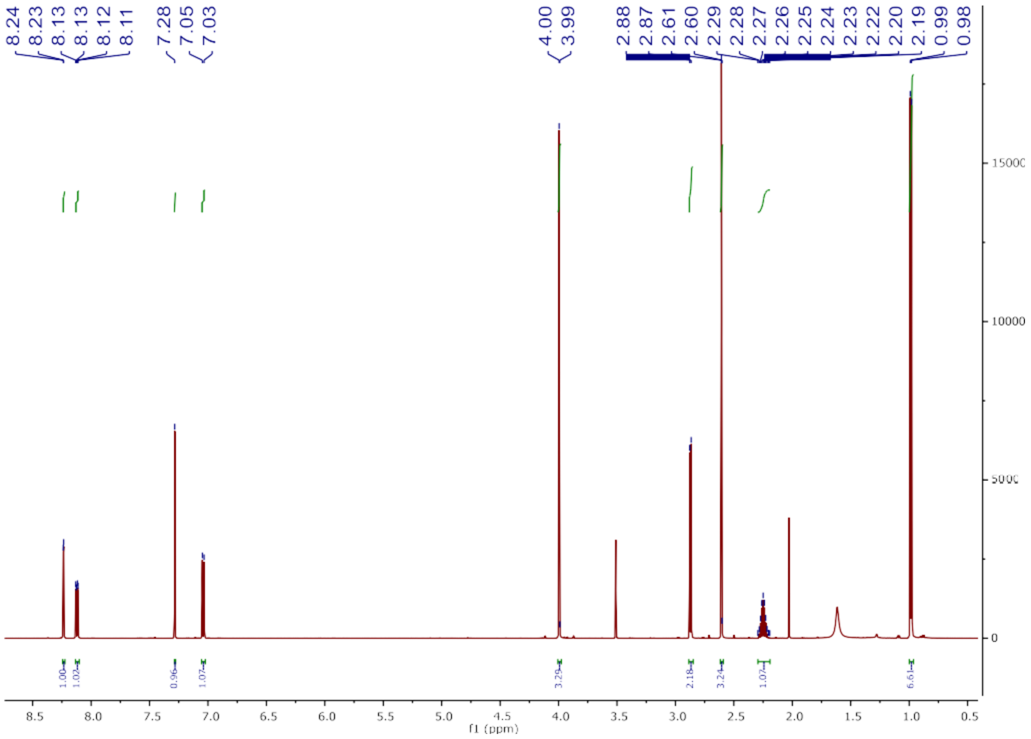

^{1}H NMR spectrum of **7'** in $CDCl_3$

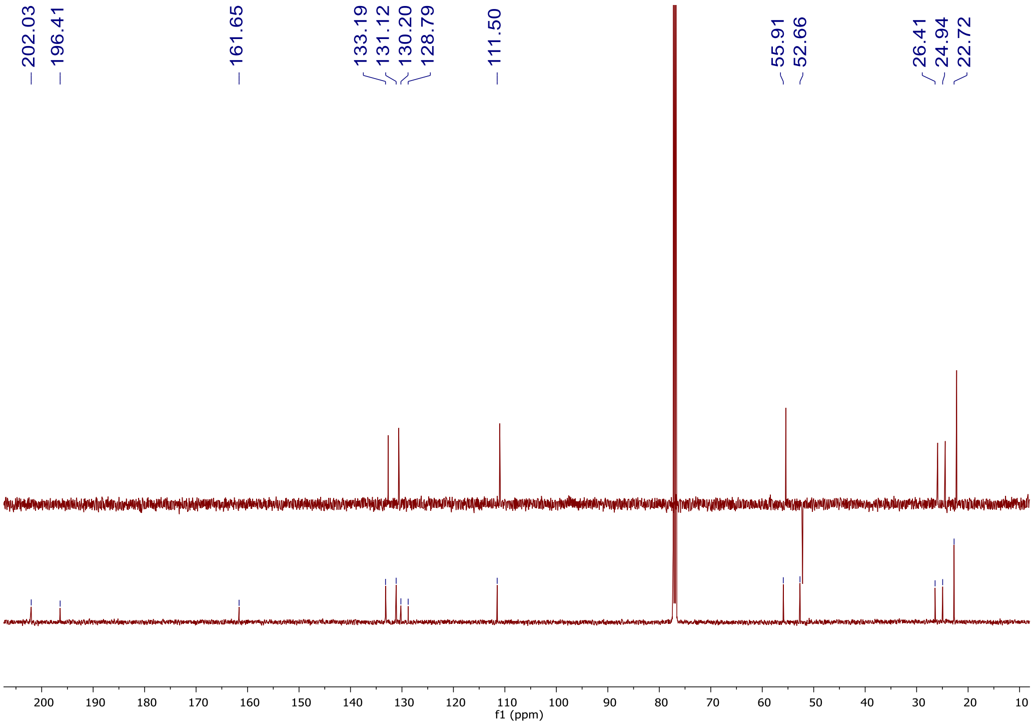

^{13}C NMR, DEPT spectrum of **7'** in $CDCl_3$

Figure S23. ^{1}H NMR, ^{13}C NMR, DEPT spectrum of **7'** in $CDCl_3$

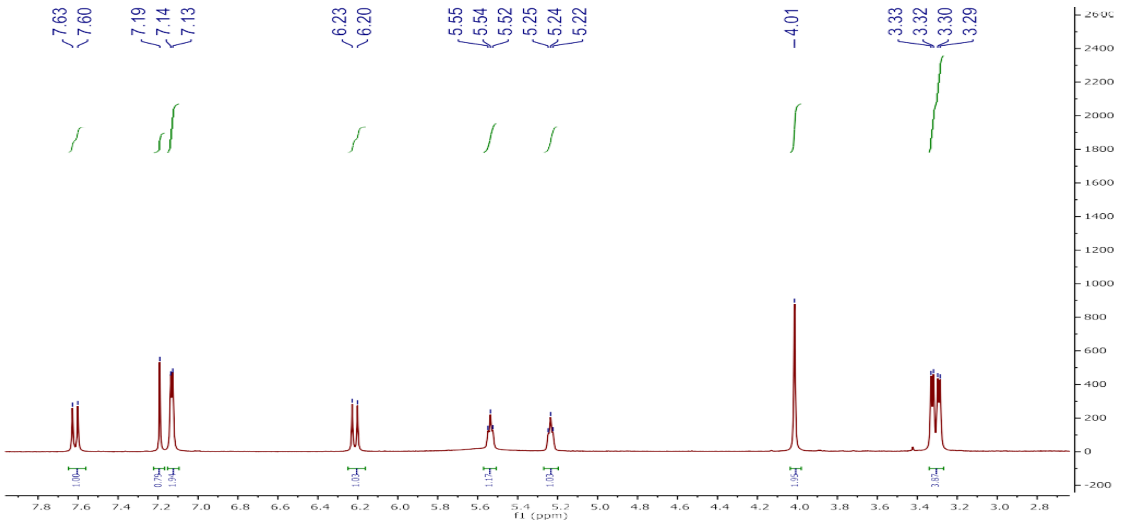

^{1}H NMR spectrum of **8’** in $CDCl_3$

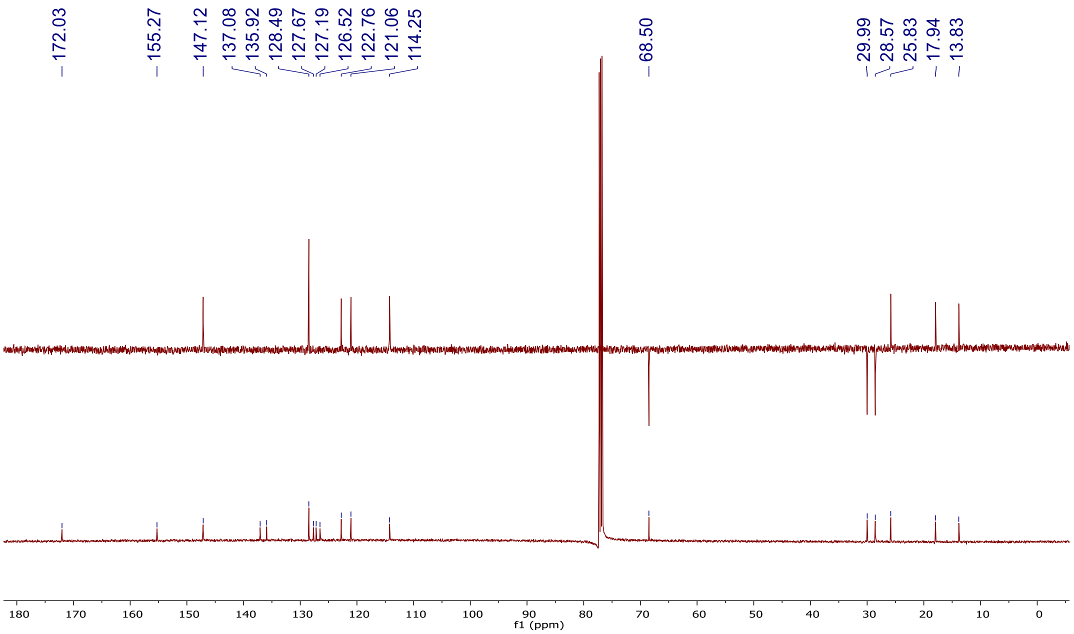

^{13}C NMR, DEPT spectrum of **8’** in $CDCl_3$

Figure S24. ^{1}H NMR, ^{13}C NMR, DEPT spectrum of **8’** in $CDCl_3$

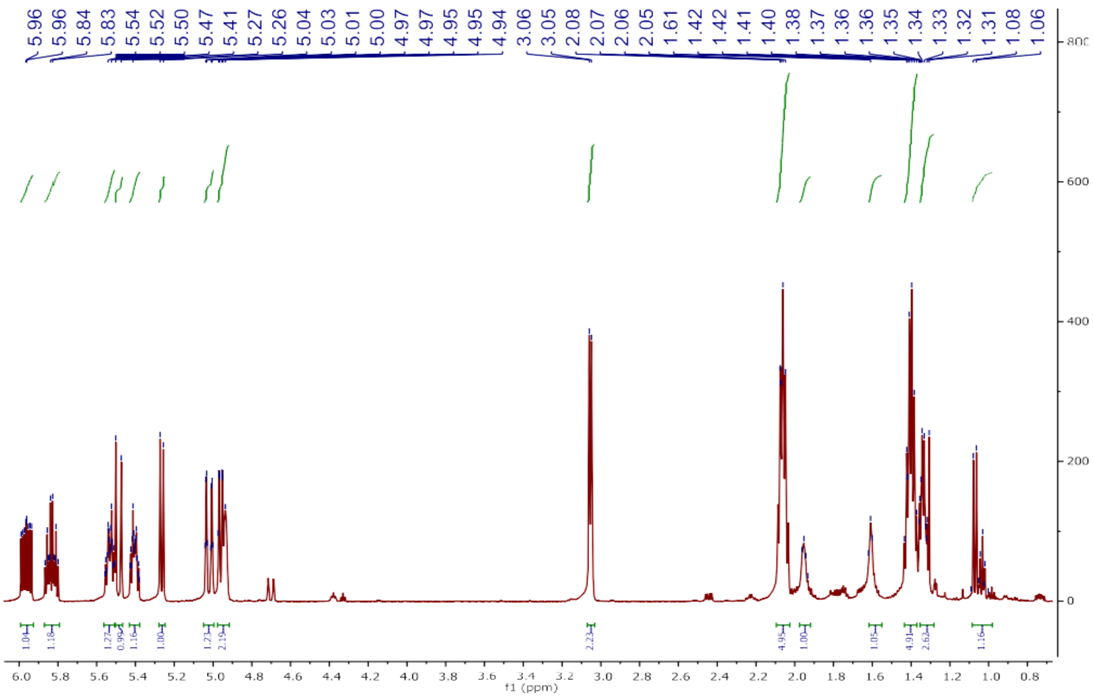

^{1}H NMR spectrum of **9’** in $CDCl_3$

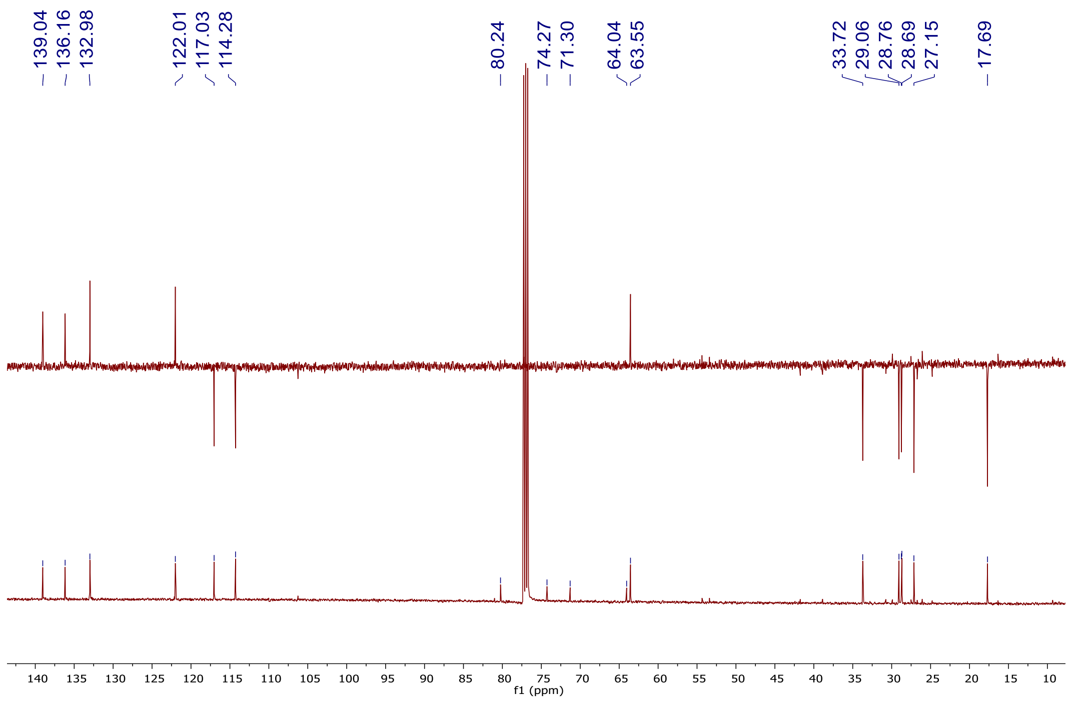

^{13}C NMR, DEPT spectrum of **9’** in $CDCl_3$

Figure S25. ^{1}H NMR, ^{13}C NMR, DEPT spectrum of **9’** in $CDCl_3$

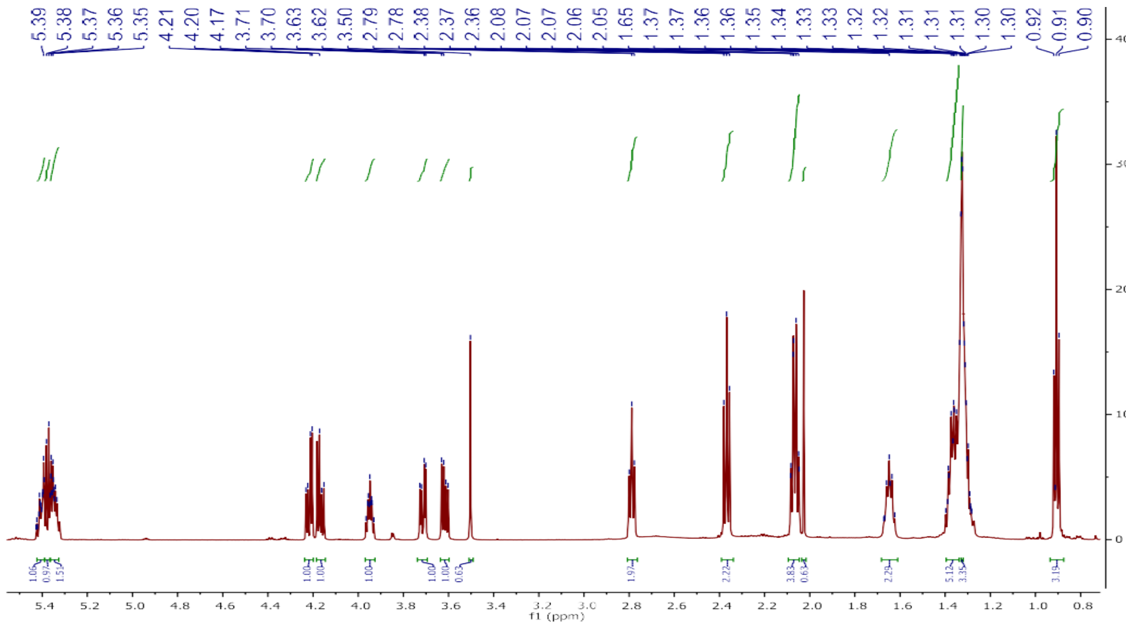

^{1}H NMR spectrum of **10'** in $CDCl_3$.

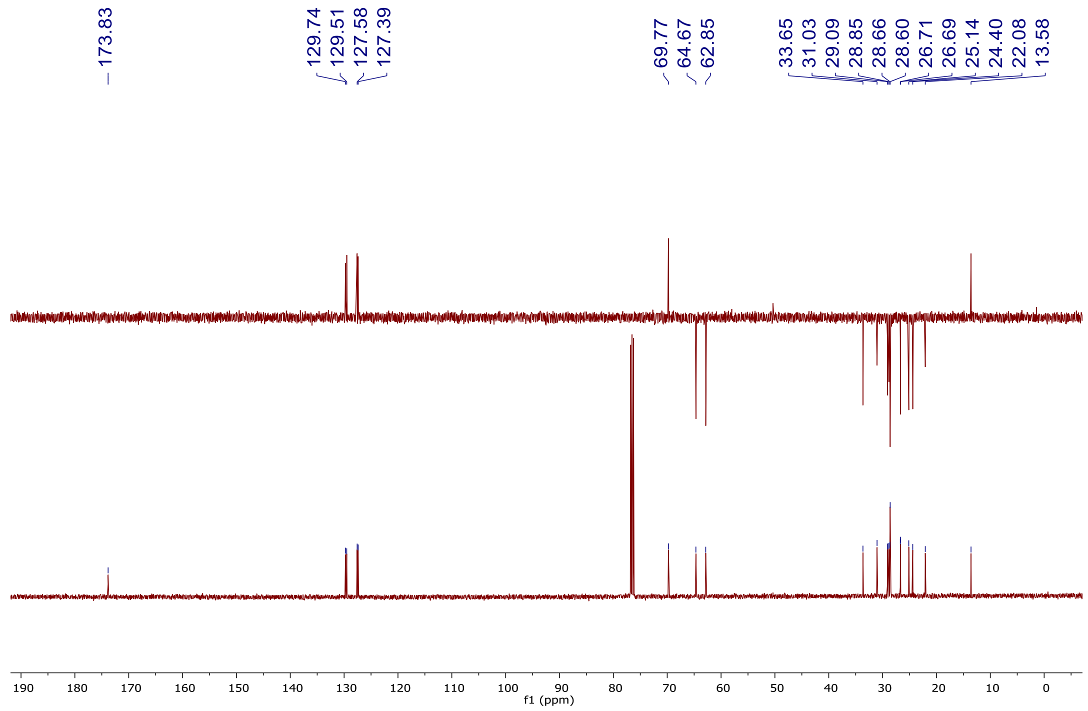

^{13}C NMR, DEPT spectrum of **10'** in $CDCl_3$.

Figure S26. ^{1}H NMR, ^{13}C NMR, DEPT spectrum of **10'** in $CDCl_3$

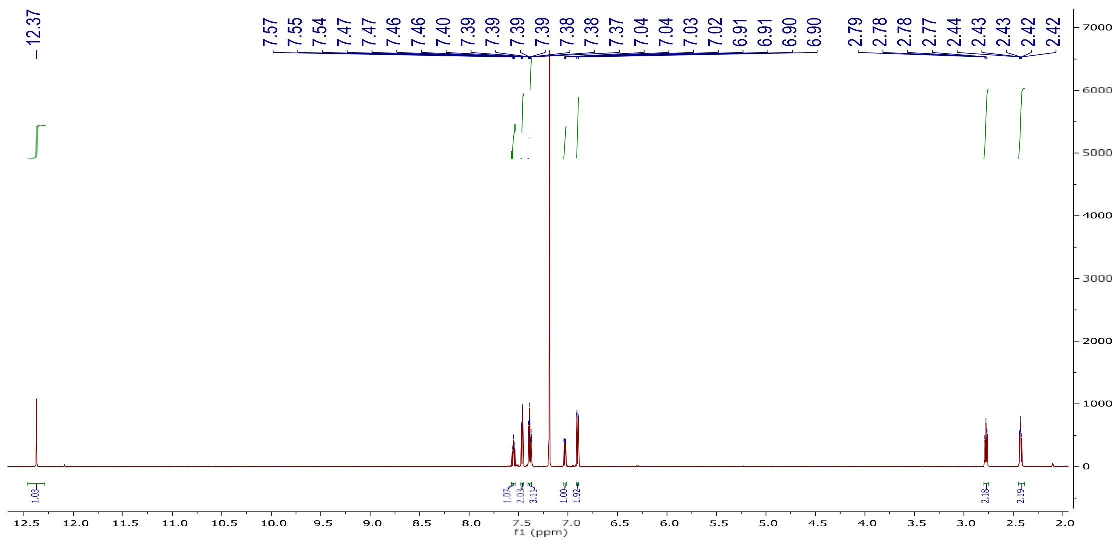

^{1}H NMR spectrum of **11’** in $CDCl_3$.

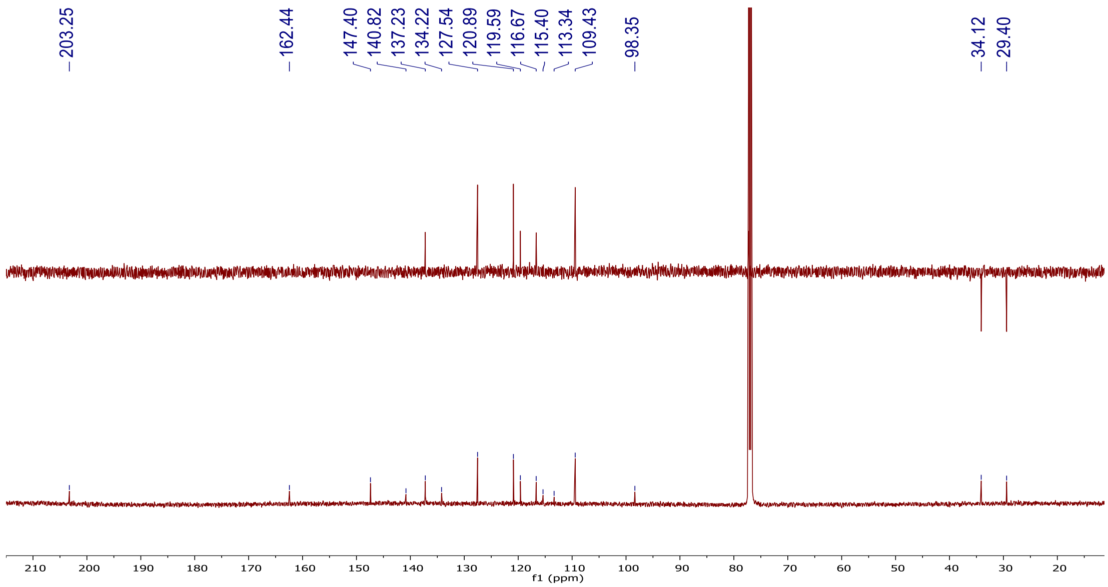

^{13}C NMR, DEPT spectrum of **11’** in $CDCl_3$.

Figure S27. ^{1}H NMR, ^{13}C NMR, DEPT spectrum of **11’** in $CDCl_3$.

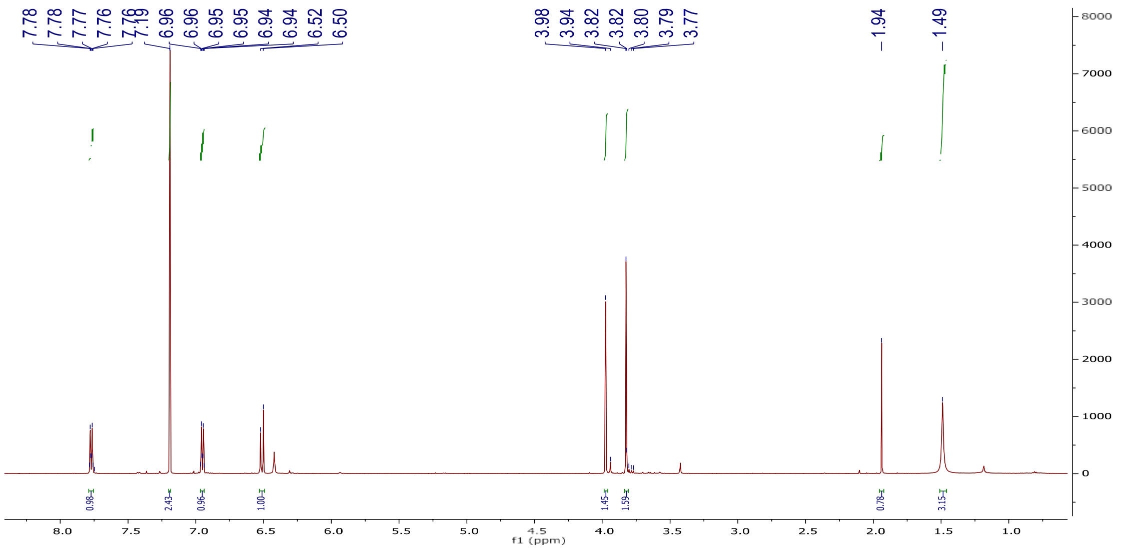

. ^{1}H NMR spectrum of **12’** in $CDCl_3$

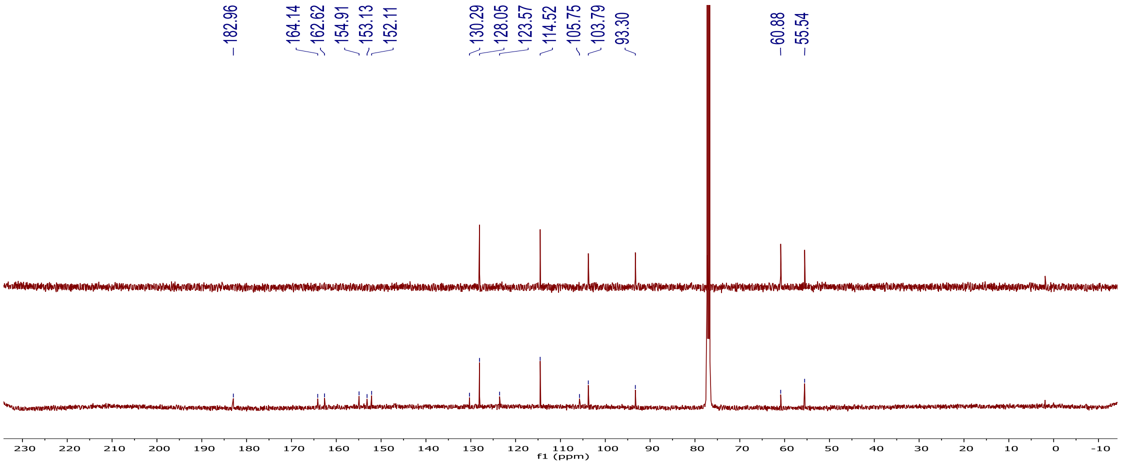

^{13}C NMR, DEPT spectrum of **12’** in $CDCl_3$.

Figure S28. ^{1}H NMR, ^{13}C NMR, DEPT spectrum of **12’** in $CDCl_3$.

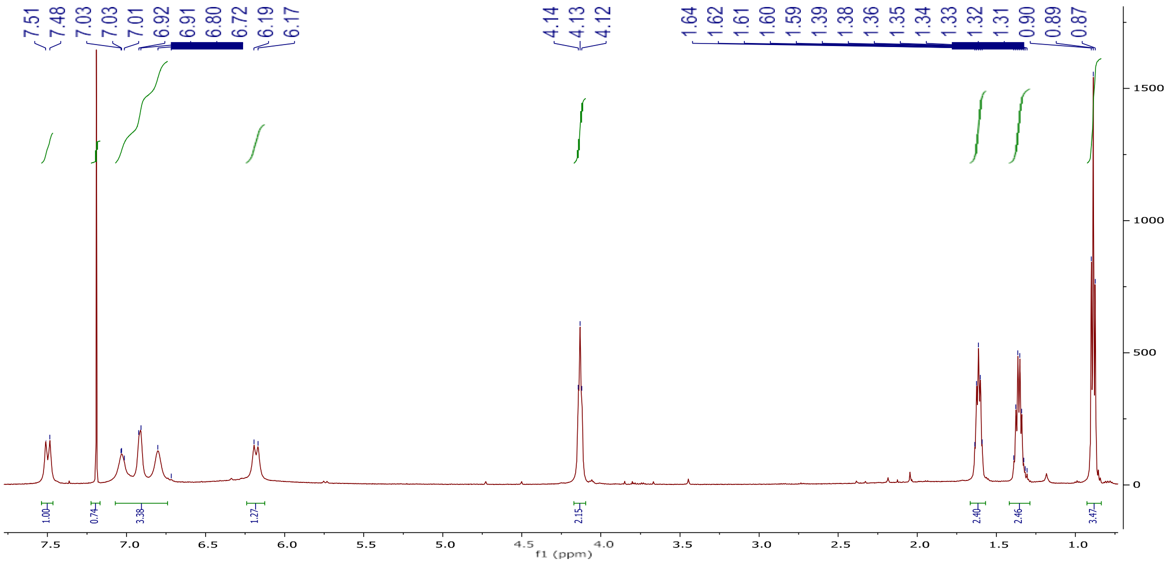

^1H NMR spectrum of **13’** in $CDCl_3$

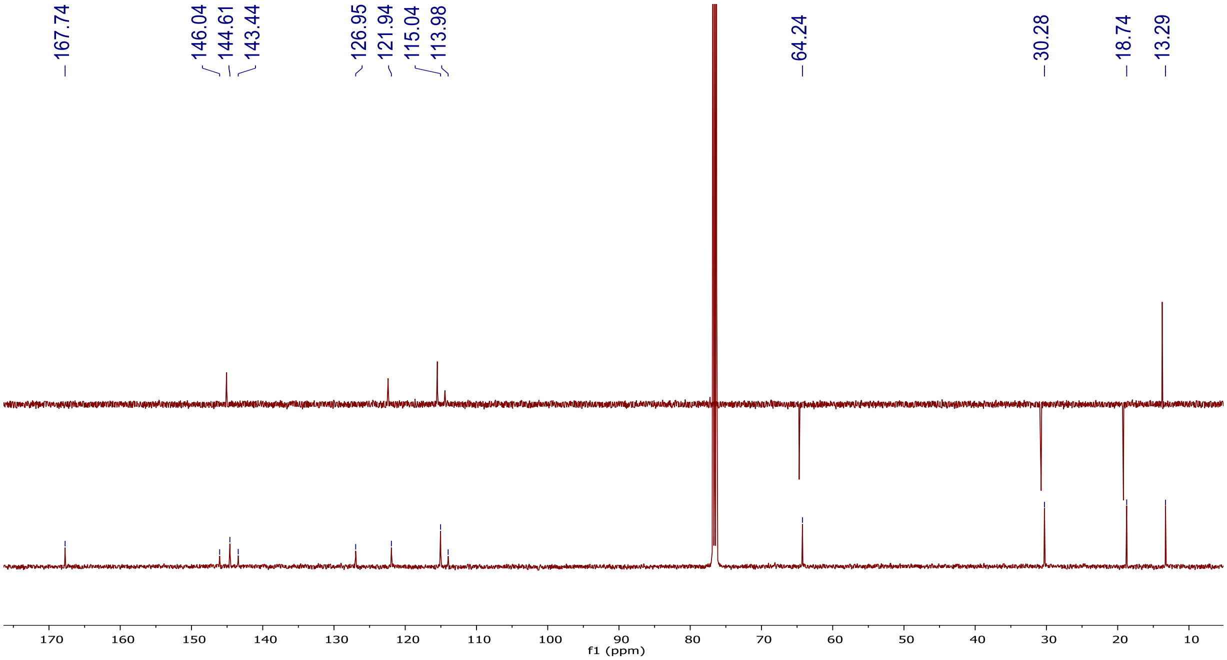

^{13}C NMR, DEPT spectrum of **13’** in $CDCl_3$

Figure S29. ^1H NMR, ^{13}C NMR, DEPT spectrum of **13’** in CDCl

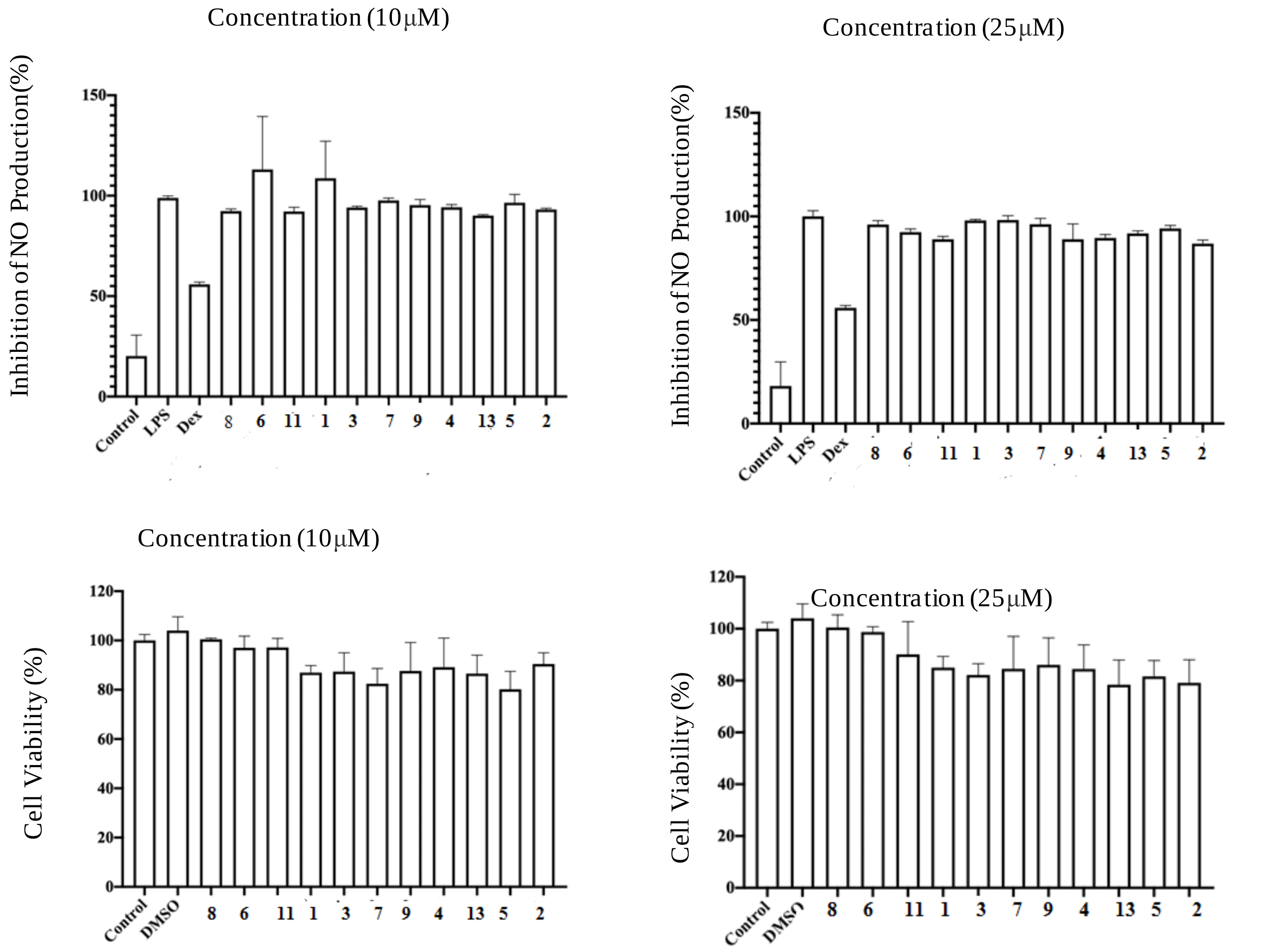

Figure S30. Inhibitory Effects of *A.nitrosa* compounds on LPS-Enhanced Inflammatory Mediators

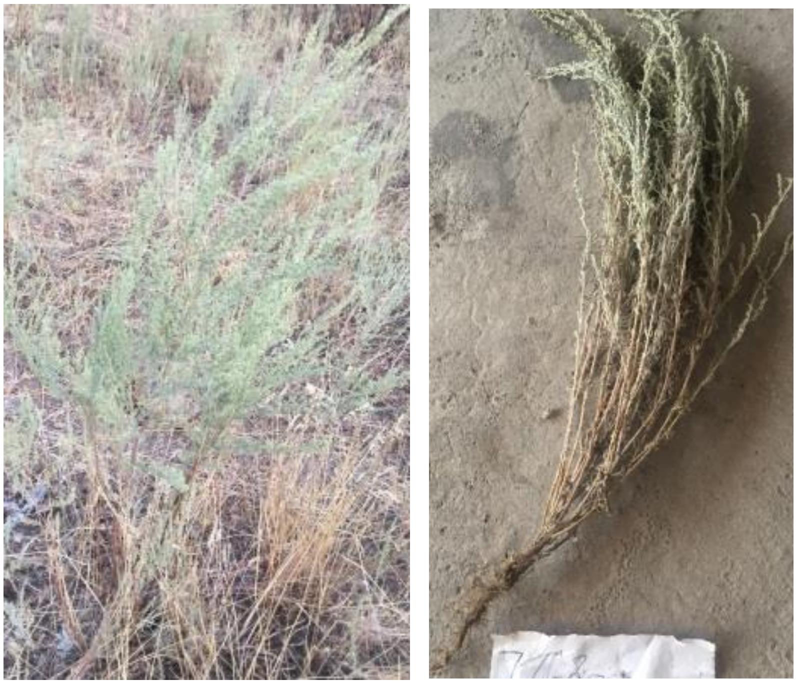

Figure S31. Pictures of the whole plant of *Artemisia nitrosa*.

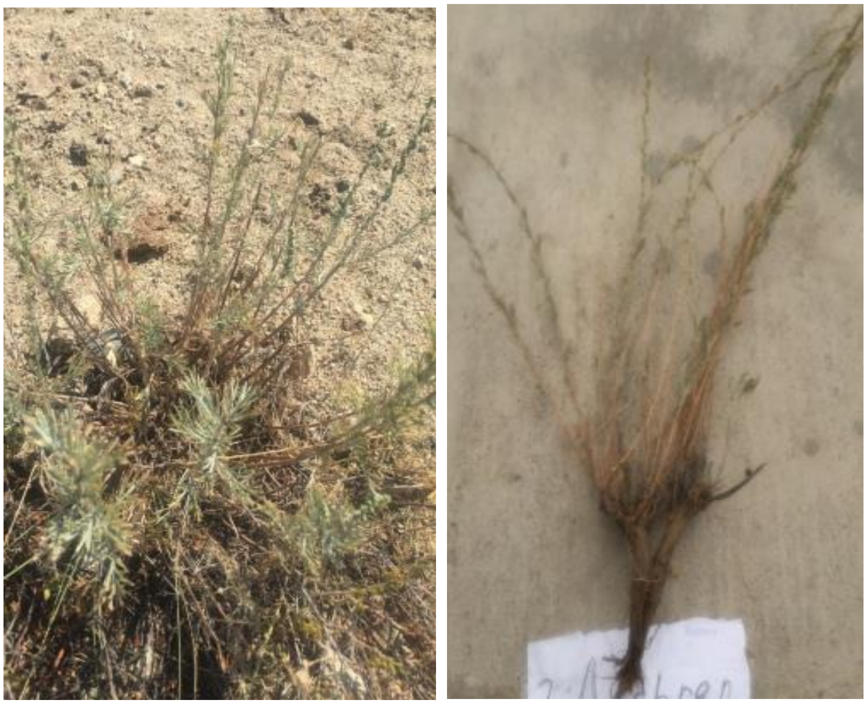

Figure S32. Pictures of the whole plant of *Artemisia marschelliana*.
